# Persistence with anti-dementia medications: a systematic review and meta-analysis

**DOI:** 10.1093/ageing/afaf151

**Published:** 2025-06-03

**Authors:** Mohammad Sistanizad, Gregory M Peterson, Tristan Ling, Mohammed Saji Salahudeen, Vyasa Murthy Akondi, Woldesellassie M Bezabhe

**Affiliations:** University of Tasmania, School of Pharmacy and Pharmacology, Hobart, Australia; Shahid Beheshti University of Medical Sciences, Faculty of Pharmacy, Tehran, Iran; University of Tasmania, School of Pharmacy and Pharmacology, Hobart, Australia; University of Tasmania, School of Pharmacy and Pharmacology, Hobart, Australia; University of Tasmania, School of Pharmacy and Pharmacology, Hobart, Australia; University of Tasmania, School of Pharmacy and Pharmacology, Hobart, Australia; University of Tasmania, School of Pharmacy and Pharmacology, Hobart, Australia

**Keywords:** dementia, anti-dementia drugs, medication persistence, systematic review, older people

## Abstract

**Background:**

Suboptimal persistence with anti-dementia drugs (ADDs) in patients with dementia is associated with poorer clinical outcomes, including accelerated disease progression, cognitive decline and increased healthcare utilisation. This study aimed to systematically review real-world persistence rates with ADDs and identify factors influencing persistence.

**Methods:**

We followed the Cochrane methodology and Preferred Reporting Items for Systematic Reviews and Meta-Analyses guidelines and searched Medline, Embase, PsycINFO and CINAHL from 1 January 1995 to 5 February 2024. Pooled persistence rates were calculated using random-effects Mantel–Haenszel models. Heterogeneity was assessed using *I*^2^ statistics, publication bias via funnel plots and Egger’s/Begg’s tests, and moderators were explored through meta-regression.

**Results:**

We included 68 studies involving 684,493 participants aged 50 years and older who received ADD. The mean 12-month persistence rate was 49% (95% CI: 42%–56%). Subgroup analyses revealed higher persistence for studies where a permissible gap for medicine refills was not required based on the methodology (67%, 95% CI: 38%–90%), those examining memantine (61%, 95% CI: 38%–82%), studies published between 2011 and 2015 (54%, 95% CI: 41%–68%) and studies conducted in Europe (57%, 95% CI: 43%–71%). Of these, the permissible gap remained an independent predictor of between-study heterogeneity in persistence (*β* = 0.36, 95% CI: 0.18–0.54).

**Conclusion:**

The meta-analysis demonstrated relatively low persistence to ADDs, which varied according to the evaluation criteria used. Targeted interventions to improve persistence with therapy may lead to better outcomes in patients with dementia. Also, a standardised framework for measuring persistence could improve research reliability.

## Key Points

- We assessed persistence with cholinesterase inhibitors and memantine using real-world data from almost 700 000 dementia patients.
- Only half of the patients remained persistent at 12 months, with persistence definition influencing study heterogeneity.
- Low persistence to anti-dementia therapy highlights the need for strategies to improve adherence and maximise treatment benefits.

## Introduction

Dementia presents a substantial challenge globally. In 2023, approximately 55 million individuals worldwide had dementia [1]. The World Alzheimer Reports projected the number of people with dementia to reach 75 million by 2030, accompanied by a cost burden estimated at US$2 trillion per annum [2, 3]. Dementia currently ranks as the seventh leading cause of death and is a significant contributor to disability and dependency among older individuals worldwide [1, 4].

Anti-dementia drugs (ADDs) currently available are cholinesterase inhibitors (ChEIs) and the N-methyl-D-aspartate receptor antagonist, memantine. These drugs are prescribed to alleviate and slow the progression of cognitive symptoms and enhance daily functioning in affected individuals, with no disease-modifying effects [5, 6]. New medicines that target amyloid plaques have recently been approved in the USA; aducanumab in 2021, lecanemab in 2023 and donanemab in 2024 [7–9]. However, evidence regarding their long-term safety and efficacy is still lacking, and their application in real-world settings remains limited.

Given this context, medications for symptomatic management of dementia continue to be the primary focus of medical treatment [10]. Nevertheless, studies conducted in various countries have indicated high discontinuation rates for ChEIs and memantine, ranging from 20% to 66%, during follow-up periods from 6 months to 5 years [11–20]. It is important to note that these rates were reported with different follow-up periods, and there were inconsistencies in the definitions of persistence and the data sources used. For example, there was variation in how studies handled patients who had passed away or were lost to follow-up.

To make effective clinical decisions, providers need data to determine whether patients have initiated pharmacotherapy and how well they are persisting with the medication regimen. Timely and accurate knowledge of adherence is critical for informing clinical decisions about the need for dose changes or interventions to address adherence behaviour. The dynamic adherence concept also supports the notion of ‘critical junctures’—periods when monitoring and supporting adherence are particularly important (e.g. early in the course of therapy, after experiencing treatment failure, following hospital discharge) [21]. Consequently, enhancing treatment adherence and persistence has become a global priority for health systems [22, 23]. Persistence with ChEIs and memantine is crucial for symptom management, slowing disease advancement, and preserving cognitive function over time [24–27]. This enhances the quality of life and prolongs survival for those affected by dementia, alleviates the burden on caregivers and the health system [28–31]. Furthermore, understanding why and how persistence varies may help develop targeted approaches to improve it.

Previously conducted reviews concerning the persistence rates associated with ADDs were published in 2017 and 2018 [32, 33]. Additionally, these reviews did not utilise a meta-analytical approach to comprehensively integrate the available data. Therefore, our primary objective was to assess 12-month persistence with ChEIs and memantine in patients with dementia in real-world practice, as reported in observational studies. In this context, ‘real-world’ refers to the use of these medications in everyday clinical settings rather than in controlled clinical trials. This approach is crucial because it provides insights into actual patient adherence, discontinuation trends, and the barriers affecting long-term treatment persistence. Such insights can help in developing targeted interventions to improve clinical outcomes in patients with dementia. Persistence rates may vary significantly depending on the definition used, particularly regarding the permissible gap between prescription refills. Furthermore, differences in persistence may exist between ChEIs and memantine, as well as across different time periods due to evolving treatment guidelines and healthcare policies. We, therefore, aimed to explore the influence of permissible refill gap, drug type, and publication year on 12-month persistence rates in our meta-regression analysis as a secondary objective.

## Methods

We followed the Cochrane methodology [34] and the Preferred Reporting Items for Systematic Reviews and Meta-Analyses (PRISMA) guidelines [35] when conducting and reporting, respectively, our systematic review. Our study was registered with PROSPERO (CRD42022361744).

### Search strategy, selection criteria and outcomes

We conducted a literature search using Medline via Ovid, Embase via Ovid, PsycINFO via Ovid, and CINAHL databases from 1 January 1995 to 5 February 2024, aligning with the global launching of ChEIs in 1995. Our search terms comprised a combination of MeSH terms and keywords related to dementia, ADDs, and persistence. The details of the search terms and results for each database are provided in Supplementary Appendix A. Furthermore, we searched Google Scholar using key concepts, manually compared the first 100 articles with retrieved references and examined the reference lists of the included articles.

We included observational studies that assessed real-world persistence with ADDs, including ChEIs, memantine, aducanumab and lecanemab. We excluded interventional studies, case reports/series, commentaries, letters, conference abstracts, theses, and editorials, along with any articles that were not published in English.

After retrieval, the studies were exported to EndNote (version 21), where duplicates were identified and removed. The remaining studies were then transferred to Covidence [36]. Two reviewers (MS and GP/MSa/TL/WB) independently conducted title and abstract screening. Studies that met the inclusion criteria at this stage underwent full-text retrieval for subsequent screening. After the full-text screening phase, two investigators independently reviewed articles and extracted relevant study data. The investigators resolved any discrepancies in data extraction through consensus. The collected data included the author’s name, country of the study, publication year, study design, sample size, age, target population, studied drugs, type of data source, definition of persistence, follow-up duration for reporting persistence, and percentage of persistent patients.

The primary outcome was the 12-month persistence rate.

### Definitions, data extraction and synthesis

We defined persistence as a dichotomous variable considering whether a patient was still taking a medicine after a predefined period [37]. For studies utilising prescription/dispensing records, such as pharmacy claim data, we recorded a permissible gap, defined as a prespecified limit on the number of days allowed between prescription refills to still be considered persistent [37]. Some studies assessed persistence using medical records to assess whether the patient was still taking the therapy without specifying a permissible gap. Therefore, we categorised the permissible gap for such studies as not required (NR). In the absence of specific guidelines recommending a standard definition for persistence, we selected a 45-day cut-off for the permissible gap to conduct subgroup analysis and meta-regression. This decision was made to approximately halve the included datapoints, facilitating a balanced comparison.

The methods for measuring and reporting persistence varied, especially regarding four subgroups of patients: deceased patients, those lost to follow-up, switchers (e.g. between different ChEIs), and those who received combination therapy (e.g. ChEI plus memantine) during the study period. To standardise reporting, we attempted to correct the documented persistence rates. Where data were available, subjects who died or were lost to follow-up during the study period were excluded from the persistence calculations. Switchers and patients who received combination therapy were considered persistent if the relevant data were provided, aligning with our objective to examine overall persistence with ADDs.

The percentage of persistent patients was calculated by dividing the number of patients continuing to use the ADD at the end of the follow-up period by the number of patients still being followed at that time. The persistence rates were inferred from studies reporting discontinuation rates; for instance, if a study reported a 25% discontinuation rate, the corresponding persistence rate was documented as 75%. We assessed persistence with ChEIs and memantine in dementia patients in real-world settings, focusing primarily on 12-month persistence and secondarily aimed to explore the influence of permissible gap, drug type, and publication year on 12-month persistence rates in our meta-regression analysis.

### Study quality assessment

Two investigators (MS and GP/MSa/TL/WB) independently assessed the quality of all included studies using the Joanna Briggs Institute (JBI) critical appraisal tool for prevalence studies [38]. Any discrepancies were resolved through consensus with a third investigator. The JBI tool consists of nine items to assess sample frame, appropriate sampling, sample size, details of setting and samples, data analysis, identification and measurement of the condition, and adequacy of the response rate. Items were structured with possible answers of ‘yes’, ‘no’, ‘unclear’ or ‘not applicable’. Based on the response to each JBI item, studies were rated as high, moderate, or low quality if they scored a total of ‘yes’ responses of ≥7, 4–6, or <4, respectively. All studies, regardless of the results of their assessed methodological quality, underwent data extraction and synthesis.

### Data synthesis and analysis

For the meta-analysis, ADDs were categorised into two groups: ChEIs and memantine. The ChEIs group included persistence with a single ChEI, two ChEIs, or all three (donepezil, rivastigmine and galantamine). For studies that reported persistence for each ChEI separately, we calculated overall ChEI persistence. Retrospective studies were included in the meta-analysis because they were likely to offer a more accurate reflection of real-world clinical practice [39]. Prospective studies, however, frequently employ strict inclusion criteria, which can result in selection bias stemming from controlled environments, and close follow-up and monitoring of participants. Consequently, the findings from these studies may have limited generalisability to real-world practice [39, 40].

The data analysis was performed using Stata/SE 18.0 (Stata Corp LLC). The pooled outcome was expressed as the proportion persistent, with 95% confidence intervals (CIs) around the summary estimate. We employed a random-effects model due to the anticipated presence of heterogeneity in the study data. The results are presented based on four subgroups: permissible gap, drug group, year of publication, and study region.

Heterogeneity between studies was examined visually by a Galbraith plot and using Cochran’s *Q*-test and the *I*^2^ statistics, with the latter describing the percentage of variation across studies [41]. The risk of publication bias was examined by visually assessing the symmetry of funnel plots. Funnel plot asymmetry was evaluated statistically using Egger’s and Begg’s tests, with a *P*-value <.05 considered suggestive of publication bias. A sensitivity analysis was performed using the leave-one-out method to assess the influence of each individual study on overall 12-month persistence rate with ChEIs and memantine. This involved systematically removing one study at a time and repeating the analysis.

A meta-regression was performed to examine the relationship between the year of publication, study region, class of ADDs, and definition of persistence (permissible gap) with persistence rate. The multivariate meta-regression model included moderators with *P*-values <.2 from univariate analyses.

**Table 1:** Characteristics of the studies included in the systematic review.

Author, year, and country	Study design, subjects	Sample size (beginning of the study)	Mean age ± SD (years)	Medications	Data source	Permissible gap (days)	Follow-up: Percentage persistent
Ahn et al., 2015, Korea [42]	Retrospective, new^a^	6461	76.4 ± 6.5	donepezil, rivastigmine, galantamine	Administrative healthcare databases	30	3 months: 50 12 months: 24
Balazs et al., 2022, Hungary [24]	Retrospective, new	donepezil 7909 rivastigmine 524 switcher 370	donepezil: 75.98 ± 7.94, rivastigmine: 76.24 ± 7.81, switchers: 75.03 ± 7.83	donepezil, rivastigmine	Administrative healthcare databases	30	12 months: donepezil: 24.6, rivastigmine: 29, switcher: 25.9 24 months: donepezil: 8.8%, rivastigmine: 11.2, switcher: 7.8 36 months: donepezil: 2.6, rivastigmine: 4, switcher: 2.2
Bent-Ennakhil et al., 2017, USA [43]	Retrospective, new	5200	81.7 ± 7.03	donepezil, rivastigmine, galantamine, memantine	Administrative healthcare databases	45	Mean 659.7 days: ADD: 78.35, donepezil: 78.06, rivastigmine: 77.66, galantamine: 72.45, memantine: 80.96
Bohlken et al., 2015, Germany [19]	Retrospective, new	12 910	79.2 ± 7.6	donepezil, rivastigmine, galantamine, memantine	Administrative healthcare databases	89	12 months: donepezil: 60.5, rivastigmine: 59.6, galantamine: 58.5, memantine: 60.5 24 months: donepezil: 51.2, rivastigmine: 50.6, galantamine: 48.6, memantine: 50.4 36 months: donepezil: 44.6, rivastigmine: 43.8, galantamine: 42.4, memantine: 44.3 48 months: donepezil: 39.0, rivastigmine: 40.3, galantamine: 38.4, memantine: 38.9 60 months: donepezil: 34.7, rivastigmine: 35.5, galantamine: 34.2, memantine: 34.4
Bohlken et al., 2017, Germany [44]	Retrospective, new	galantamine:2442 donepezil: 5669 memantine: 4416 oral rivastigmine: 642 patch rivastigmine: 2334	Mean age ranged from 78.0 ± 7.7 to 80.6 ± 8.0	donepezil, rivastigmine, galantamine, memantine	Administrative healthcare databases	180	12 months: galantamine: 8 mg: 77.5, 16 mg: 83.9, 24 mg: 83.2 donepezil: 5 mg: 78.8, 10 mg: 81.0 memantine: 10 mg: 76.9, 20 mg: 94.7 rivastigmine oral: 1.5 mg: 79.6, 3 mg: 70.0, 4.5 mg: 79.2, 6 mg: 77.6 rivastigmine patch: 4.6 mg: 77.0, 9.5 mg: 77.5
Byun et al., 2022, Korea [45]	Retrospective, new	8653	76.4 ± 8.2	donepezil, rivastigmine, galantamine, memantine	Registry databases	NA	12 months: ADD 47, donepezil: 48.2, rivastigmine: 39.5, galantamine: 39.5, memantine: 49.8
Clerici et al., 2009, Italy [46]	Prospective, new	451	77 ± 7.4	memantine	Medical records including patients’ visits	NR	6 months: 83.3
Dybicz et al., 2006, USA [47]	Retrospective, new	2873	95.8% ≥ 65 years	donepezil, rivastigmine, galantamine	Administrative healthcare databases	29	12 months: donepezil: 56.9 rivastigmine: 53.8 galantamine: 53.0
Zheng Kang et al. 2019, Singapore [48]	Prospective, new	144	76.9 ± 7.9	donepezil, rivastigmine, galantamine, memantine	Medical records including patients’ visits	60	12 months: 79.3
Osada et al., 2018, Japan [49]	Retrospective, new	312	82.0 ± 6.0	rivastigmine patch	Medical records including patients’ visits	NR	168 days: 62.2
Pariente et al. 2012, Canada [50]	Retrospective, new	24 394	≥66	donepezil, rivastigmine, galantamine	Administrative healthcare databases	42	12 months: 66.3
Taipale et al., 2014, Finland [13]	Retrospective, new	6858	79.3 ± 6.70	donepezil, rivastigmine, galantamine, memantine	Registry databases	NA	12 months: ChEIs: 80.01, memantine: 87.79
Amuah et al., 2010, Canada [51]	Retrospective, new and pretreated^b^	1080	79.8 ± 7.1	donepezil, rivastigmine, galantamine	Administrative healthcare databases	60	191 days (permissible gap = 60): 50 12 months (permissible gap = 60): 33.6 40 months (permissible gap = 60):16 40 months (permissible gap = 90): 20.7 40 months (permissible gap = 120): 23.7
Borah et al., 2010, USA [52]	Retrospective, new	3091	79.81 ± 8.25	donepezil, rivastigmine, galantamine, memantine	Administrative healthcare databases	60	12 months: 59.8
Brewer et al., 2013, Ireland [11]	Retrospective, new	14 197	>70	donepezil, rivastigmine, galantamine, memantine	Administrative healthcare databases	63	6 months: 69.9 12 months: 56.2
Fisher et al., 2017, Canada [53]	Retrospective, new	24 526	80.1 ± 8.3	donepezil, rivastigmine, galantamine	Administrative healthcare databases	30, 90	12 months (permissible gap = 90): ChEIs: 51, donepezil: 54, galantamine: 57, rivastigmine oral: 31, rivastigmine patch: 41 12 months (permissible gap = 30): ChEIs: 43, donepezil: 45, galantamine: 49, rivastigmine oral: 26, rivastigmine patch: 32
Fisher et al., 2016, Canada [54]	Retrospective, new	45 537	79.5 ± 8.2	donepezil, rivastigmine, galantamine	Administrative healthcare databases	90	12 months: 49
Fukuda et al., 2022, Japan [15]	Retrospective, new	20 474	82.2 ± 6.3	donepezil, rivastigmine, galantamine, memantine	Administrative healthcare databases	60	1 month: 89.1 3 months: 79.4 6 months: 72.6 12 months: 64.5 18 months: 58.3
Gardette et al., 2014, 12 European countries [14]	Prospective, new	557	76.6 ± 7.2	donepezil, rivastigmine, galantamine	Medical records including patients’ visits	35	24 months: 52.5
Haider et al., 2014, Austria [12]	Retrospective, new	15 809	79.9 ± 7.7	donepezil, rivastigmine, galantamine, memantine	Administrative healthcare databases	90	6 months: ADD: 66.0, donepezil: 63.5, rivastigmine: 62.8, galantamine: 69.8, memantine: 73.0 12 months: ADD: 41.5, donepezil: 40.0, rivastigmine: 32.7, galantamine: 45.3, memantine: 55.0
Herrmann et al., 2009, Canada [55]	Prospective, new	5622	>65	donepezil, rivastigmine, galantamine	Administrative healthcare databases	A gap of >30 days or total number of missed days allowed per year >120 days	12 months: galantamine ER 53.6, donepezil 45.9, rivastigmine: 40.2
Hermann et al., 2007, Canada [56]	Prospective, new	28 961	80.3 ± 6.4	donepezil, rivastigmine, galantamine	Administrative healthcare databases	120	Average length of follow-up was 823 days for patients residing exclusively in the community, 1021 days for those residing exclusively in long-term care, and 1010 days for those dwelling in the community at the initiation of ChEI therapy: 31.1
Kogut et al, 2005,'USA [57]	Retrospective, new	1183	82.4	donepezil, rivastigmine, galantamine	Administrative healthcare databases	30	6 months: 73.7
Kongpakwattana et al., 2019 Thailand [17]	Retrospective, new	698	78.1 ± 7.8	donepezil, rivastigmine, galantamine, memantine	Medical records including patients’ visits	30	12 months: ADD: 21.1, ChEIs: 21.17, memantine: 20.71
Kröger et al., 2010, Netherlands [58]	Retrospective, new	3369	76.3 ± 7.7	donepezil, rivastigmine, galantamine	Registry databases	30	1 month: 91.5 6 months: 69.2 36 months: 41.0
Ku et al., 2018, Taiwan [28]	Retrospective, new	8614	Approx. 74 years	donepezil, rivastigmine, galantamine	Administrative healthcare databases	90	12 months: 60 24 months: 42.5
Le Couteur et al., 2012, Australia [59]	Retrospective, new	18 598	Men: median age of 81 (IQR:76–85) Women: median age of 79 (IQR:75–84)	donepezil, rivastigmine, galantamine	Administrative healthcare databases	99	6 months: 70 12 months: 54.7 24 months: 43 36 months: 32.9
Minthon et al., 2009, Sweden [60]	Prospective, new	217	73.7 ± 7.6	rivastigmine	Medical records including patients’ visits	NR	24 months: 73
Nakagawa et al., 2017, Japan [61]	Prospective, new	661	96% ≥ 65	galantamine	Medical records including patients’ visits	NR	3 months: 80 12 months: 60 18 months: 53.3
Nazir et al., 2010, UK [62]	Prospective, new	30	100% > 52 years	rivastigmine transdermal patch	Medical records including patients’ visits	NR	6 months: 88.9
Olchanski, 2023, USA [63]	Retrospective, new	1343	82	donepezil, rivastigmine, galantamine, memantine	Registry databases	45	12 months: 76.4
Tu et al., 2015, China [64]	Retrospective, new and pre-treated	88	78.9 ± 8.96	memantine	Medical records including patients’ visits	NR	6 months: 46.6
Umegaki et al., 2008, Japan [65]	Retrospective, new	264	79.6 ± 6.5	donepezil	Medical records including patients’ visits	NR	Mean 231.4 ± 145.6 days: 64.2
Park et al., 2021, Asia [66]	Prospective, new	398	75.46 ± 7.10	donepezil	Medical records including patients’ visits	NR	12 months: 81.82
Ndukwe et al., 2015, New Zealand [67]	Retrospective, new	1999	79.5 ± 6.4	donepezil	Administrative healthcare databases	31	6 months: 65 12 months: 51 18 months: 41 24 months: 33 36 months: 17
Kadohara et al., 2017, Japan [68]	Retrospective, new	group 1: 28 581 group 2: 75 011	group 1: 79.6 ± 7.4 group 2: 80.9 ± 7.3	donepezil, rivastigmine, galantamine, memantine	Administrative healthcare databases	60	12 months: group 1: 59.5, group 2: 58.5
Gill et al., 2004, Canada [69]	Retrospective, new	6424	80.3	donepezil	Administrative healthcare databases	180	7 months: 72.2
Olazaran et al., 2013, Spain [25]	Prospective, new	240	77 ± 6.3	donepezil, rivastigmine, galantamine	Medical records including patients’ visits	NR	12 months: 78.33 24 months: 77.93 36 months: 77.32 48 months: 76.86 60 months: 79.46
Pariente et al., 2010, France [70]	Retrospective, new	942	79.6	donepezil, rivastigmine, galantamine	Administrative healthcare databases	60	12 months: 45.3
Thorpe et al., 2016, USA [71]	Retrospective, new	3481	76% > 65	donepezil, rivastigmine, galantamine, memantine	Administrative healthcare databases	30	12 months: ADD: 33.85, ChEIs: 39.83, memantine: 40.82
Suh et al., 2005, USA [72]	Retrospective, new	783	79.23 ± 6.28	donepezil or rivastigmine	Administrative healthcare databases	30, 60, 90	12 months (permissible gap = 30): 50.45 12 months (permissible gap = 60): 59.00 12 months (permissible gap = 90): 63.35
Steininger et al., 2020, Germany and UK [20]	Retrospective, new	Germany: 3863 UK: 3342	Germany: 80.9 ± 1.7 UK: 81.9 ± 1.7	donepezil, rivastigmine, galantamine, memantine	Registry databases	90	12 months: Germany: 55.2 UK: 80.2
Saleh et al., 2013, Canada [73]	Prospective, new	63	74.56 ± 7.78	donepezil, rivastigmine, galantamine	Medical records including patients’ visits	NR	6 months: 69.8
Abughosh et al., 2008, USA [74]	Retrospective, new	1564	83.1 ± 9.1	donepezil, rivastigmine, galantamine	Administrative healthcare databases	180	12 months: 57.3 24 months: 15.2
Rungsanpanya et al., 2012, Thailand [75]	Retrospective, new	80	80.6 ± 7.0	donepezil, rivastigmine, galantamine, memantine	Medical records including patients’ visits	NR	12 months: ADD: 83.3, ChEIs: 73.9, memantine: 87.5
Seibert et al., 2012, Germany [76]	Prospective, new: 638, pre-treated: 457	1113	76.5 ± 7.7	transdermal rivastigmine	Medical records including patients’ visits	NR	4 months: 84.1
Lai et al., 2016, Taiwan [77]	Prospective, new:174, pre-treated: 127	301	77.6 ± 7.19	donepezil, rivastigmine, galantamine, memantine	Medical records including patients’ visits	NR	6 months: 87.4
Maclagan et al., 2018, Canada [78]	Retrospective, pre-treated	17 560	83.9 ± 6.3	donepezil, rivastigmine, galantamine	Administrative healthcare databases	30	12 months: 23.2
Mador et al., 2003, Australia [79]	Prospective, new	64	78.4	donepezil, rivastigmine, galantamine	Medical records including patients’ visits	NR	Mean follow up 13 weeks: 84
Kim et al., 2024, South Korea [80]	Retrospective, new	3997	84.4 ± 8.0	donepezil, rivastigmine, galantamine, memantine	Medical records including patients’ visits	NR	Cohort 1: 300.2 ± 312.0 days Cohort 2: 349.8 ± 316.1 days 75.6
Hsieh et al., 2021, Taiwan [81]	Prospective, new and pre-treated	108	77.2 ± 9.0	rivastigmine oral solution	Medical records including patients’ visits	NR	12 months: 82
Mossello et al., 2004, Italy [82]	Prospective, new and pre-treated	407	78.2 ± 0.3	donepezil, rivastigmine, galantamine	Registry databases	NA	9 months: 52.1
Olazaran et al., 2023, Spain [83]	Retrospective, new and pre-treated	4747	94.6% ≥ 70	donepezil, rivastigmine, galantamine, memantine	Medical records including patients’ visits	NR	36 months: donepezil: 54.0, memantine: 36.2, rivastigmine: 43.5, galantamine: 48.8
Niznik et al., 2019, USA [84]	Retrospective, pre-treated	37 106	75% > 80	donepezil, rivastigmine, galantamine	Administrative healthcare databases	30	Up to 11 months: 69.8
Lim et al., 2018, Korea [85]	Retrospective, new	groups: old (≥85 years): 77 younger (<85): 78	Old: 87.34 ± 2.49 Younger: 74.62 ± 3.47	donepezil, rivastigmine, galantamine, memantine	Medical records including patients’ visits	NR	24 months: Total: 78.1 Old: 83.1 Young: 73.1
Chang, 2019, Taiwan [86]	Prospective, new:143, pre-treated: 5	148	74.4 ± 7.7	oral rivastigmine	Medical records including patients’ visits	14	12 months: 75.2
Frankfort et al., 2005, Netherland [87]	Retrospective, new	154	78.4 ± 5.8	rivastigmine	Medical records including patients’ visits	NR	6 months: 58.2 12 months: 40 24 months: 28.1 36 months: 2.6
Roe et al., 2002, US [88]	Retrospective, new	59	77.3	donepezil	Administrative healthcare databases	14	6 months: 61.02
Chang et al., 2021, Taiwan [89]	Prospective, pre-treated	285	78.1 ± 7.7	rivastigmine patch	Medical records including patients’ visits	NR	11 months (48 weeks): 71.7
Van Der Putt et al., 2006, UK [90]	Prospective, new	1322	79.9 ± 7.2	donepezil, rivastigmine, galantamine	Registry databases	NA	4 months: 76.2
Wallin et al., 2007, Sweden [91]	Prospective, new	435	74.6 ± 6.5	donepezil	Registry databases	NA	2 months: 96.7 6 months: 93.6 12 months: 89.0 18 months: 81.4 24 months: 75.9 30 months: 67.7 36 months: 56.7
Sonde et al., 2013, Sweden [92]	Retrospective, new	241	81.1 ± 6.0	donepezil, rivastigmine, galantamine, memantine	Medical records including patients’ visits	NR	24 months: 91.02
Cagnin et al., 2015, Italy [93]	Prospective, pre-treated	174	Approx. 78 years	rivastigmine patch	Medical records including patients’ visits	NR	6 months: 79.9
Matthews et al., 2000, UK [94]	Prospective, new	80	75	donepezil	Medical records including patients’ visits	NR	3 months: 91.2 6 months: 55 9 months: 45 12 months: 36.25 18 months: 15
Stamouli et al., 2011, Greece [95]	Prospective, new:1690, pre-treated: 880	2570	74.8 ± 6.8	memantine	Medical records including patients’ visits	NR	3 months: 94.36 6 months: 77.53
Wurm et al., 2020, Austria [96]	Retrospective, new and pre-treated	127 896	Median 82.3 [IQR 76.77, 86.62]	donepezil, rivastigmine, galantamine, memantine	Administrative healthcare databases	NA	Median 13.3 months: 73.8
Vidal et al., 2008, France [97]	Retrospective, new: 1976, pre-treated: 3307	5283	80.8 ± 7.6	memantine	Administrative healthcare databases	NA	6 months: 74 12 months: 64
Kostev et al., 2019, Poland [16]	Retrospective, new	66,030	95% ≥ 65 years	donepezil, rivastigmine, galantamine, memantine	Registry databases	90	12 months: donepezil: 42.2, rivastigmine: 46.0, memantine: 65.9

ADD, anti-dementia drug; ChEI, cholinesterase inhibitor; NA, not applicable; NR, not required.

^a^New: means newly initiated with ADD which was defined as patients who had either never had previous ADD therapy or categorised as newly diagnosed and/or initiated ADD or patients who had not been on the currently prescribed ADD at least within the past 6 months (Kogut et al., 2005, USA [57] 2 months, Chang, 2019, Taiwan [86] 4 weeks)

^b^Pretreated: defined as previously treated with any kind of ADD.

## Results

### Overview of the included studies

**Figure 1:**
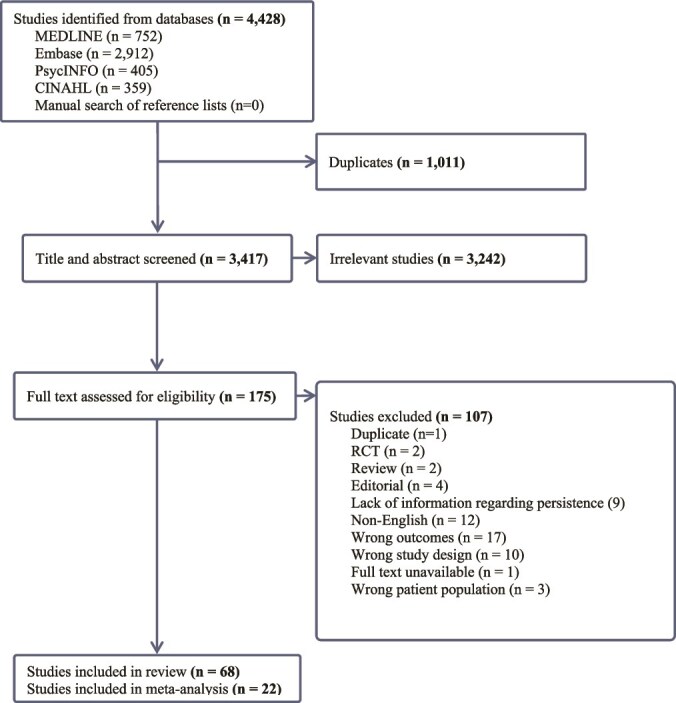
PRISMA flow diagram of the study selection process.

A total of 4428 records were identified through our literature searches. After removing duplicates, 3417 records were assessed. Following title and abstract screening, 175 records were deemed eligible for full-text evaluation. We included 68 studies involving 684 493 participants. The PRISMA flow diagram of the study selection process is shown in Figure 1. Characteristics of the included studies are shown in Table 1. Twenty-five studies (37%) were from Europe [11–13, 16, 19, 24, 25, 44, 46, 58, 60, 62, 70, 76, 82, 83, 87, 90–97], followed by 19 (28%) from North American sites [43, 47, 50–57, 63, 69, 71–74, 78, 84, 88], 18 (26%) from Asia [15, 17, 28, 42, 45, 48, 49, 61, 64, 65, 68, 75, 77, 80, 81, 85, 86, 89], 3 (4%) from Australia and New Zealand [59, 67, 79], and 3 (4%) from multiple regions [14, 20, 66]. There were 45 (66%) retrospective [11–13, 15–17, 19, 20, 24, 28, 42–45, 47, 49–54, 57–59, 63–65, 67–72, 74, 75, 78, 80, 83–85, 87, 88, 92, 96, 97] and 23 (34%) prospective [14, 25, 46, 48, 55, 56, 60–62, 66, 73, 76, 77, 79, 81, 82, 86, 89–91, 93–95] studies. The studies exhibited a wide range of sample sizes, ranging from 30 [62] to 127,896 [96], with a mean of 10,066 and a standard deviation of 22,050. Although our search strategy initially included terms for aducanumab and lecanemab, no studies reporting persistence data for these medications met our inclusion criteria.

In 53 studies, participants who were newly started on ADDs were included [11–17, 19, 20, 24, 25, 28, 42–50, 52–62, 65–75, 79, 80, 83, 85, 87, 88, 90–92, 94]. Four studies specifically focused on individuals who had been previously treated with any form of ADD [78, 84, 89, 93], while 11 studies encompassed both newly initiated and pre-treated subjects [51, 64, 76, 77, 81–83, 86, 95–97]. Thirty-one studies utilised ‘administrative healthcare databases’ [11, 12, 15, 19, 24, 28, 42–44, 47, 50–57, 59, 67–72, 74, 78, 84, 88, 96, 97], 28 studies used ‘medical records including patients’ visits’ [14, 17, 25, 46, 48, 49, 60–62, 64–66, 73, 75–77, 79–81, 83, 85–87, 89, 92–95], and nine studies employed ‘registry databases’ [13, 16, 20, 45, 58, 63, 82, 90, 91]. In 25 studies, defining a permissible gap was deemed unnecessary [25, 46, 49, 60–62, 64–66, 73, 75–77, 79–81, 83, 85, 87, 89, 91–95], while in 6 studies where it was necessary, the authors did not mention it [13, 45, 82, 90, 96, 97]. The permissible gap ranged from 14 [86, 88] to 180 days [44, 69, 74]. The duration of follow-up ranged from less than 3 months [15, 42, 91, 94, 95] to 60 months [19, 25]. The most commonly reported follow-up periods were 12 months (40 studies) [11–13, 15–17, 19, 20, 24, 25, 28, 42, 44, 45, 47, 48, 50–55, 59, 61, 63, 66–68, 70–72, 74, 75, 78, 81, 86, 87, 91, 94, 97], 6 months (19 studies) [11, 12, 15, 46, 57–59, 62, 64, 67, 73, 77, 87, 88, 91, 93–95, 97], and 24 months (13 studies) [14, 19, 24, 25, 28, 59, 60, 67, 74, 85, 87, 91, 92].

### Quality assessment of included studies

The quality of the studies overall was rated as high, with a mean (standard deviation [SD]) score of 7.34 (1.58). Of the 68 studies, 50 were considered to be of high quality (scoring 7/9 or higher) [11–17, 19, 20, 24, 25, 28, 42–47, 50–55, 57–61, 63, 66–74, 77, 78, 81–84, 88, 93, 95–97], 16 were of moderate quality (scoring 4–6) [48, 49, 56, 62, 64, 65, 75, 76, 80, 86, 87, 89–92, 94], and two were rated as poor quality (scoring <4) [79, 85]. Supplementary Appendix B summarises the JBI quality assessment scores for each study.

### Meta-analysis

Twenty-eight data points from 22 retrospective studies [16, 17, 19, 24, 28, 42, 44, 47, 50, 51, 53, 54, 59, 67, 68, 70–72, 74, 75, 78, 87] examining 12-month persistence with ChEIs/memantine were included in the meta-analysis. Only one study [87] provided data that enabled adjustments to the persistence rate. Six data points from 4 studies [16, 17, 70, 74] lacked information on deceased individuals and those lost to follow-up, while in 10 data points from eight studies [19, 24, 42, 44, 50, 59, 67, 68] deceased patients were classified as non-persistent without adequate data for adjustment. Detailed information on how the included data points addressed deceased individuals, lost-to-follow-up subjects, combination therapy, and medication switches, as well as the ability to adjust persistence rates, are provided in Supplementary Appendix C, which contains the total number of subjects, and the number of persistent subjects reported for each data point.

**Figure 2:**
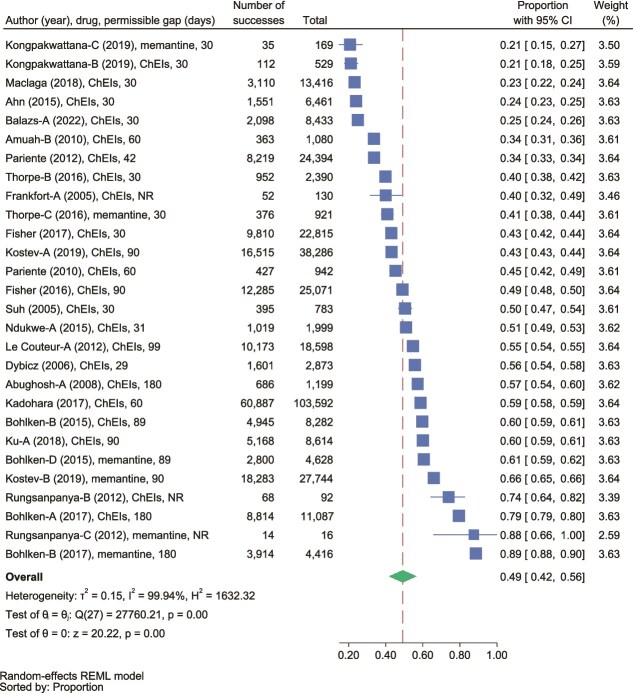
Forest plot: pooled proportion of 12-month persistence with ChEIs and memantine at 12 months; ChEI: cholinesterase inhibitor, NR: not required.

A random-effects meta-analysis yielded an overall 12-month persistence rate of 49% (95% CI: 42%–56%) (Figure 2). High heterogeneity was observed (*Q* < 0.001, *I*^2^ = 99.94%). The funnel plot was symmetrical, suggesting no significant publication bias (Supplementary Appendix D, Egger’s *P* = .241; Begg’s *P* = .767). Leave-one-out sensitivity analyses (Supplementary Appendix E) showed that excluding one outlier [44] reduced the persistence rate to 47% (95% CI: 47%–54%). This specific data point was also recognised as an outlier based on the Galbraith plot (Supplementary Appendix F). Subgroup analyses (Table 2, Supplementary Appendix G) revealed higher persistence for studies where a permissible gap was not required based on their methodology (67%, 95% CI: 38%–90%), those using memantine (61%, 95% CI: 38%–82%), studies published between 2011 and 2015 (54%, 95% CI: 41%–68%), and studies conducted in Europe (57%, 95% CI: 43%–71%).

**Table 2:** Subgroup analysis of 12-month persistence based on permissible gap, drug group, publication year, and country region.

			Tests for heterogeneity	
Analysis group	No. of data points	Pooled persistent proportion (95% CI)	*P*-value (*Q* statistics)	*I* ^2^%
**Permissible gap**				
≤45 days [16, 19, 28, 44, 51, 54, 59, 68, 70, 74]	12	0.35 (0.28, 0.43)	<.001	99.78
>45 days [17, 24, 42, 47, 50, 53, 67, 71, 72, 78]	13	0.59 (0.50, 0.67)	<.001	99.94
NR [75, 87]	3	0.67 (0.38, 0.90)	<.001	93.85
**Anti-dementia drug type**				
ChEIs [16, 17, 19, 24, 28, 42, 44, 47, 50, 51, 53, 54, 59, 67, 68, 70–72, 74, 75, 78, 87]	22	0.46 (0.39, 0.53)	<.001	99.92
Memantine [16, 17, 19, 44, 71, 75]	6	0.61 (0.38, 0.82)	<.001	99.90
**Publication Year**				
2005–2010 [47, 51, 70, 72, 74, 87]	6	0.47 (0.40, 0.55)	<.001	97.24
2011–2015 [19, 42, 50, 59, 67, 75]	8	0.54 (0.41, 0.68)	<.001	99.90
2016–2022 [16, 17, 28, 44, 53, 54, 68, 71, 78]	14	0.47 (0.35, 0.59)	<.001	99.97
**Country Region**				
Asia and Oceania [17, 28, 42, 59, 67, 68, 75]	9	0.49 (0.33, 0.65)	<.001	99.94
Europe [16, 19, 24, 44, 70, 87]	9	0.57 (0.43, 0.71)	<.001	99.95
North America [47, 50, 51, 53, 54, 71, 72, 74, 78]	10	0.42 (0.36, 0.49)	<.001	99.75

CI, confidence interval; NR, not required; ChEI, cholinesterase inhibitor.

The mean 12-month persistence rate for ChEIs and memantine across prospective studies [25, 48, 55, 61, 66, 81, 91, 94, 98] was 71% (95% CI: 59%–82%) (Supplementary Appendix H). Additionally, mean persistence rates for these treatments were analysed over various follow-up durations: 4–7 months, 24 months, and 36 months. The reported data consisted of 8 [49, 51, 57, 58, 64, 69, 88, 90], 3 [24, 28, 74], and 7 [19, 25, 58, 59, 67, 87] data points, with corresponding mean persistence rates of 65% (95% CI: 57%–72%), 21% (95% CI: 5%–43%), and 31% (95% CI: 18%–46%) (Supplementary Appendix I), respectively.

### Meta-regression

According to the meta-regression analysis (Table 3), the permissible gap (equal to or less than 45 days, more than 45 days, and NR) (*β* = 0.36, 95% CI: 0.18–0.54, *P* < .001) surfaced as an independent moderator of heterogeneity among studies, with higher persistence rates observed in studies that defined a permissible gap of more than 45 days and those for which a permissible gap was not required based on their methodology. The type of medication (ChEIs versus memantine) was not identified as a factor contributing to the heterogeneity among studies regarding the persistence rate (*β* = 0.23, 95% CI: −0.05 to 0.52, *P* = .112).=

**Table 3:** Meta-regression analysis to explore the specific moderators impacting the between-study heterogeneity of 12-month persistence.

Moderator	Category 1	Category 2	Category 3	Regression coefficient (95% CI)	*P*-value	*I* ^2^ inconsistency *Q* statistic
Year	2005–2010 [47, 51, 70, 72, 74, 87]	2011–2015 [19, 42, 50, 59, 67, 75]	2016–2022 [16, 17, 28, 44, 53, 54, 68, 71, 78]	−0.02 (−0.20, −0.17)	.860	99.94%; 26 525.72 (26 df), *P* < .001
Region	Asia and Oceania [17, 28, 42, 59, 67, 68, 75]	Europe [16, 19, 24, 44, 70, 87]	North America [47, 50, 51, 53, 54, 71, 72, 74, 78]	−0.07 (−0.25, −0.11)	.458	99.93%; 21 975.56 (26 df), *P* < .001
Medications	ChEIs [16, 17, 19, 24, 28, 42, 44, 47, 50, 51, 53, 54, 59, 67, 68, 70–72, 74, 75, 78, 87]	Memantine [16, 17, 19, 44, 71, 75]	—	0.29 (−.06, 0.64)	.110	99.93%; 23 188.27 (26 df), *P* < .001
Permissible Gap	≤45 days [16, 19, 28, 44, 51, 54, 59, 68, 70, 74]	>45 days [17, 24, 42, 47, 50, 53, 67, 71, 72, 78]	NR [75, 87]	0.37 (0.19, 0.56)	<.001	99.90%; 13 461.28 (26 df), *P* < .001
**Multivariable**						
Permissible Gap	≤45 days [16, 19, 28, 44, 51, 54, 59, 68, 70, 74]	>45 days [17, 24, 42, 47, 50, 53, 67, 71, 72, 78]	NR [75, 87]	0.36 (0.18, −0.54)	<.001	99.89%; 11 279.11 (df 25). *P* < .001
Medications	ChEIs [16, 17, 19, 24, 28, 42, 44, 47, 50, 51, 53, 54, 59, 67, 68, 70–72, 74, 75, 78, 87]	Memantine [16, 17, 19, 44, 71, 75]	—	0.23 (−0.05, 0.52)	.112	

CI, confidence interval; ChEIs, cholinesterase inhibitors; NR, not required.

## Discussion

This systematic review evaluated 684 493 users of ChEIs and memantine, finding that only 50% remained persistent at 12 months. Meta-regression highlighted the permissible gap as an independent moderator of heterogeneity, with more flexible criteria associated with higher persistence rates. A non-significant trend indicated slightly greater persistence with memantine compared to ChEIs. Our findings demonstrated varying persistence rates across follow-up periods, with higher rates at 4–7 months (65%) compared to 24 months (21%) and 36 months (31%). The 35% discontinuation rate during the early phase (4–7 months) may be attributed to a lack of perceived benefit, which could influence patients’ motivation to continue treatment [18], while the decline in persistence at 24–36 months is likely influenced by limited or uncertain ongoing therapeutic efficacy, and potential adverse effects of these medications, particularly in advanced stages of dementia [99, 100]. The decision to withdraw ADD is typically made on an individual basis, considering the patient’s well-being and the perspectives of families or caregivers. Additionally, uncertainties persist regarding the long-term benefits and potential adverse effects of these medications, particularly in severe dementia [63, 101].

Our examination indicated a superior mean 12-month persistence rate for ChEIs and memantine in prospective studies, at 71% compared to 49% in retrospective studies. Patients in prospective studies or registries may have been more likely to persist with treatment due to closer and more regular monitoring, better access to care, or greater awareness of the benefits of treatment [39, 102]. To represent real-world practice and treatment persistence more accurately, we included retrospective studies in our assessment and meta-analysis.

In this study, the highest rate of treatment persistence was noted among studies that did not require a permissible gap in medication persistence (NR group), with 67% of subjects continuing their treatment after 12 months. The absence of a standardised definition for permissible gaps represents a critical methodological issue, as it influences reported persistence rates and contributes to heterogeneity across studies. While the 45-day cut-off used in our analysis was arbitrary, our findings demonstrated that the presence or absence of a defined permissible gap significantly impacts persistence rates. This underscores the importance of establishing a clinically meaningful and standardised definition for permissible gaps in research. Adopting consistent methodologies in future studies will enhance the reliability, comparability, and interpretability of persistence data.

Consistent with our findings, in a study conducted by Simard et al. [103], the persistence of oral antidiabetic medications was evaluated using different definitions for permissible gaps, specifically at 1.5 and 2 times the duration of the prior prescription. The researchers concluded that increasing the permissible gap to 2 times the duration of the previous prescription raised the 6-month persistence rate from 62% to 71%. This trend was also evident at the 12-month follow-up, where the persistence rate improved from 51% to 62%.

We observed a higher mean persistence rate for memantine (61%) compared to ChEIs (46%); however, this difference was not statistically significant in the multivariable model. Haider et al. reported that patients on memantine had a greater persistence rate than those on ChEIs, with 55% of memantine users still taking the medication after 12 months, compared to only 33% of those using rivastigmine [12]. This disparity may be attributed to the adverse effects commonly associated with ChEIs, such as gastrointestinal side effects, neuropsychiatric events, and potential cardiac adverse effects [12, 104–106]. In contrast, memantine exhibits a more favourable safety and tolerability profile [107].

Studies reported several factors that may influence persistence with ADDs, encompassing the severity of dementia, educational attainment, medication burden, economic standing, treatment practices and adverse drug reactions [14–16, 18, 20, 32, 33, 53, 66, 98]. Fukuda et al. [15] found that female participants and those with a higher number of co-treatments (excluding ADDs) were less likely to discontinue treatment. Additionally, an increased need for long-term care was associated with a higher incidence of treatment discontinuation. In another study, Bohlken et al. [19] found that younger age, male gender, having heart failure or hypertension, and possessing private health insurance were associated with a lower risk of discontinuation. Additionally, patients treated by nurse practitioners were less likely to stop their medication compared to those under the care of general practitioners. Contrary to the findings of Fukuda et al. [15], the Bohlken study [19] found that the number of co-treatments was not associated with a higher persistence rate, while also reporting a higher persistence rate among male patients.

A 12-month persistence rate of only 49% raises significant concerns regarding the long-term management of dementia. Persistence with ADD is crucial for symptom management and preserving cognitive function over time [24–27]. Furthermore, previous reviews indicated that treatment with ChEIs lowered the risk of mortality associated with dementia by a quarter during follow-up conducted between 6 and 36 months [30, 31]. Given the poor persistence reported here, further research investigating targeted interventions to promote persistence with ADDs is warranted. These could involve strategies such as enhanced patient and carer education programs addressing concerns about side effects and collaborative care models that integrate pharmacists and other healthcare professionals to provide ongoing support and medication management. Future research should focus on rigorously evaluating the effectiveness and cost-effectiveness of these interventions in diverse real-world settings.

While our research offers important insights into the rates of persistence with ADDs, several limitations need to be recognised. First, limiting our review to English-language publications may have led to publication bias. Second, discrepancies in the definitions of persistence and variability across various studies hinder our ability to compare all articles comprehensively. Third, the absence of pertinent data concerning patient mortality, attrition rates, modifications in therapeutic interventions (including alterations in pharmacological agents or the initiation of combination therapies) during follow-up, and planned discontinuation due to disease progression, constituted another considerable limitation that constrained our ability to calculate the persistence rate in accordance with our established criteria. A fourth limitation is the inconsistent reporting of detailed clinical and demographic information in the included studies. Although large insurance and administrative databases often contain comprehensive data, the original studies did not consistently provide essential variables such as the type of dementia, time since diagnosis, and comorbid conditions. In particular, while some studies reported baseline demographic data (e.g. age and sex at study initiation), they generally did not include this information at the end of follow-up for subjects who either discontinued treatment or remained persistent. This shortfall hindered our ability to assess the potential confounding effects of these parameters on treatment persistence.

## Conclusion

The meta-analysis revealed that over half of patients discontinued their anti-dementia therapy within 1 year of starting treatment, with rates varying based on the evaluation criteria used. The significant heterogeneity observed across studies likely reflects, in part, the influence of various definitions of persistence. A standardised framework for measuring persistence would contribute to more reliable research findings, while treatment outcomes in dementia may benefit from targeted interventions to promote persistence with these drugs.

## Supplementary Material

aa-24-2572-File002_afaf151

## Data Availability

Further information is available upon request from the corresponding author.

## References

[1] Dementia Key Facts. World Health Organization. 2024. https://www.who.int/news-room/fact-sheets/detail/dementia (31 January 2024, date last accessed).

[2] Alzheimer’s Disease International World Alzheimer Report 2010, the Global Economic Impact of Dementia. Alzheimer’s Disease International (ADI); 2010:56. https://www.alzint.org/u/WorldAlzheimerReport2010.pdf. (31 January 2024, date last accessed).

[3] Alzheimer’s Disease International , World Alzheimer Report 2015 the Global Impact of Dementia an Analysis of Prevalence, Incidence, Cost and Trends. Alzheimer’s Disease International (ADI); 2015:87. https://www.alzint.org/u/WorldAlzheimerReport2015.pdf. (31 January 2024, date last accessed).

[4] Livingston G, Huntley J, Liu KY et al. Dementia prevention, intervention, and care: 2024 report of the lancet standing commission. Lancet 2024;404:572–628. 10.1016/S0140-6736(24)01296-0. 39096926

[5] 2021 Alzheimer's disease facts and figures. Alzheimers Dement 2021;17:327–406. 10.1002/alz.12328. 33756057

[6] Miculas DC, Negru PA, Bungau SG et al. Pharmacotherapy evolution in Alzheimer’s disease: current framework and relevant directions. Cells 2022;12. 10.3390/cells12010131. PMC9818415 36611925

[7] Administration USFD . BLA 761178. Alzheimer’s Association. 2024. Updated 07/06/2021. https://www.accessdata.fda.gov/drugsatfda_docs/appletter/2021/761178Orig1s000ltr.pdf. (06 August 2024, date last accessed).

[8] Administration USFD . BLA 761269. U.S. Food & Drug Administration. 2024. Updated 06/01/2021. https://www.accessdata.fda.gov/drugsatfda_docs/appletter/2023/761269Orig1s000ltr.pdf. (06 August 2024, date last accessed).

[9] Administration USFD . BLA 761248. U.S. Food & Drug Administration. 2024. Updated 02/07/2024. https://www.accessdata.fda.gov/drugsatfda_docs/appletter/2024/761248Orig1s000ltr.pdf. (06 August 2024, date last accessed).

[10] Hafiz R, Alajlani L, Ali A et al. The latest advances in the diagnosis and treatment of dementia. Cureus 2023;15: e50522. 10.7759/cureus.50522. 38222245 PMC10787596

[11] Brewer L, Bennett K, McGreevy C et al. A population-based study of dosing and persistence with anti-dementia medications. Eur J Clin Pharmacol 2013;69:1467–75. 10.1007/s00228-013-1483-y. 23443628

[12] Haider B, Schmidt R, Schweiger C et al. Medication adherence in patients with dementia: an Austrian cohort study. Alzheimer Dis Assoc Disord 2014;28:128–33. 10.1097/wad.0000000000000006. 24113561

[13] Taipale H, Tanskanen A, Koponen M et al. Antidementia drug use among community-dwelling individuals with Alzheimer's disease in Finland: a nationwide register-based study. Int Clin Psychopharmacol 2014;29:216–23. 10.1097/YIC.0000000000000032. 24608822 PMC4047310

[14] Gardette V, Lapeyre-Mestre M, Piau A et al. A 2-year prospective cohort study of antidementia drug non-persistency in mild-to-moderate Alzheimer's disease in Europe: predictors of discontinuation and switch in the ICTUS study. CNS Drugs 2014;28:157–70. 10.1007/s40263-013-0133-3. 24408842

[15] Fukuda H, Maeda M, Murata F et al. Anti-dementia drug persistence following donepezil initiation among Alzheimer's disease patients in Japan: LIFE study. J Alzheimers Dis 2022;90:1177–86. 10.3233/JAD-220200. 36213993 PMC9741733

[16] Kostev K, Kurylo P, Kosik J et al. One-year persistence with donepezil, memantine, and rivastigmine in more than 66,000 elderly patients followed in Poland. J Alzheimers Dis 2019;70:899–905. 10.3233/JAD-190508. 31306136

[17] Kongpakwattana K, Dilokthornsakul P, Dejthevaporn C et al. Compliance and persistence with Alzheimer's disease treatment: a retrospective analysis of multiregional hospital databases in Thailand. J Med Econ 2019;22:26–34. 10.1080/13696998.2018.1534739. 30303420

[18] Maxwell CJ, Stock K, Seitz D et al. Persistence and adherence with dementia pharmacotherapy: relevance of patient, provider, and system factors. Can J Psychiatry 2014;59:624–31. 10.1177/070674371405901203. 25702361 PMC4304581

[19] Bohlken J, Weber S, Rapp MA et al. Continuous treatment with antidementia drugs in Germany 2003-2013: a retrospective database analysis. Int Psychogeriatr 2015;27:1335–42. 10.1017/s1041610215000654. 25899936

[20] Steininger G, Kostev K. The role of the treating practice in persistence among dementia patients in Germany and the UK. Int J Clin Pharmacol Ther 2020;58:247–53. 10.5414/CP203670. 32213286

[21] Menditto E, Cahir C, Malo S et al. Persistence as a robust indicator of medication adherence-related quality and performance. Int J Environ Res Public Health 2021;18. 10.3390/ijerph18094872. PMC8124987 34063641

[22] Stirratt MJ, Curtis JR, Danila MI et al. Advancing the science and practice of medication adherence. J Gen Intern Med 2018;33:216–22. 10.1007/s11606-017-4198-4. 29204969 PMC5789101

[23] Menditto E, Orlando V, De Rosa G et al. Patient centric pharmaceutical drug product design-the impact on medication adherence. Pharmaceutics 2020;12. 10.3390/pharmaceutics12010044. PMC7023035 31947888

[24] Balazs N, Bereczki D, Ajtay A et al. Cholinesterase inhibitors for the treatment of dementia: real-life data in Hungary. Geroscience 2022;44:253–63. 10.1007/s11357-021-00470-7. 34655009 PMC8811017

[25] Olazarán J, Navarro E, Rojo JM. Persistence of cholinesterase inhibitor treatment in dementia: insights from a naturalistic study. Dement Geriatr Cogn Dis Extra 2013;3:48–59. 10.1159/000345279. 23637699 PMC3617973

[26] Rountree SD, Chan W, Pavlik VN et al. Persistent treatment with cholinesterase inhibitors and/or memantine slows clinical progression of Alzheimer disease. Alzheimers Res Ther 2009;1:7. 10.1186/alzrt7. 19845950 PMC2874259

[27] Mauskopf JA, Paramore C, Lee WC et al. Drug persistency patterns for patients treated with rivastigmine or donepezil in usual care settings. J Manag Care Pharm 2005;11:231–51. 10.18553/jmcp.2005.11.3.231. 15804207 PMC10438117

[28] Ku L-JE, Li C-Y, Sun Y. Can persistence with cholinesterase inhibitor treatment lower mortality and health-care costs among patients with Alzheimer’s disease? A population-based study in Taiwan. Am J Alzheimers Dis Other Demen 2018;33:86–92. 10.1177/1533317517734639. 29210284 PMC10852471

[29] Chen S, Price AC, Cardinal RN et al. Association between antidementia medication use and mortality in people diagnosed with dementia with Lewy bodies in the UK: a retrospective cohort study. PLoS Med 2022;19:e1004124. 10.1371/journal.pmed.1004124. 36472984 PMC9725132

[30] Truong C, Recto C, Lafont C et al. Effect of cholinesterase inhibitors on mortality in patients with dementia: a systematic review of randomized and nonrandomized trials. Neurology 2022;99:e2313–25. 10.1212/WNL.0000000000201161. 36096687

[31] Havreng-Théry C, Oquendo B, Zolnowski-Kolp V et al. Cholinesterase inhibitors and memantine are associated with a reduced mortality in nursing home residents with dementia: a longitudinal observational study. Alzheimers Res Ther 2024;16:117. 10.1186/s13195-024-01481-0. 38812028 PMC11134888

[32] El-Saifi N, Moyle W, Jones C et al. Medication adherence in older patients with dementia: a systematic literature review. J Pharm Pract 2018;31:322–34. 10.1177/0897190017710524. 28539102

[33] Smith D, Lovell J, Weller C et al. A systematic review of medication non-adherence in persons with dementia or cognitive impairment. PloS One 2017;12:e0170651. 10.1371/journal.pone.0170651. 28166234 PMC5293218

[34] JPT H, Thomas J, Chandler J et al. (eds). *Cochrane Handbook for Systematic Reviews of Interventions*. 6.5 edition, 2024.

[35] Page MJ, McKenzie JE, Bossuyt PM et al. The PRISMA 2020 statement: an updated guideline for reporting systematic reviews. BMJ 2021;372:n71. 10.1136/bmj.n71. 33782057 PMC8005924

[36] Covidence Systematic Review Software. Veritas Health Innovation. 2024. www.covidence.org.

[37] Cramer JA, Roy A, Burrell A et al. Medication compliance and persistence: terminology and definitions. Value Health 2008;11:44–7. 10.1111/j.1524-4733.2007.00213.x. 18237359

[38] Munn Z, Moola S, Lisy K et al. Chapter 5: systematic reviews of prevalence and incidence. In: Aromataris E, Munn Z (eds.), *JBI Manual for Evidence Synthesis*, 2020.

[39] Claeys MJ, Beauloye C, Pourbaix S et al. Real world insights on the initiation and treatment duration of oral antiplatelets in acute coronary syndromes: a retrospective cohort study. Eur Heart J-Cardiovasc Pharmacother 2017;3:189–97. 10.1093/ehjcvp/pvw043. 28122793 PMC5843130

[40] van Onzenoort HA, Menger FE, Neef C et al. Participation in a clinical trial enhances adherence and persistence to treatment: a retrospective cohort study. Hypertension 2011;58:573–8. 10.1161/HYPERTENSIONAHA.111.171074. 21825228

[41] Higgins JP, Thompson SG, Deeks JJ et al. Measuring inconsistency in meta-analyses. BMJ 2003;327:557–60. 10.1136/bmj.327.7414.557. 12958120 PMC192859

[42] Ahn SH, Choi NK, Kim YJ et al. Drug persistency of cholinesterase inhibitors for patients with dementia of Alzheimer type in Korea. Arch Pharm Res 2015;38:1255–62. 10.1007/s12272-014-0500-8. 25336105

[43] Bent-Ennakhil N, Coste F, Xie L et al. A real-world analysis of treatment patterns for cholinesterase inhibitors and memantine among newly-diagnosed Alzheimer's disease patients. Neurol Ther 2017;6:131–44. 10.1007/s40120-017-0067-7. 28508250 PMC5447560

[44] Bohlken J, Jacob L, Kostev K. Association between anti-dementia treatment persistence and daily dosage of the first prescription: a retrospective analysis in neuropsychiatric practices in Germany. J Alzheimers Dis 2017;58:37–44. 10.3233/JAD-170091. 28372337

[45] Byun J, Lee DY, Jeong CW et al. Analysis of treatment pattern of anti-dementia medications in newly diagnosed Alzheimer's dementia using OMOP CDM. Sci Rep 2022;12:4451. 10.1038/s41598-022-08595-1. 35292697 PMC8924152

[46] Clerici F, Vanacore N, Elia A et al. Memantine in moderately-severe-to-severe alzheimers disease: a postmarketing surveillance study. Drugs Aging 2009;26:321–32. 10.2165/00002512-200926040-00003. 19476399

[47] Dybicz SB, Keohane DJ, Gary Erwin W et al. Patterns of cholinesterase-inhibitor use in the nursing home setting: a retrospective analysis. Am J Geriatr Pharmacother 2006;4:154–60. 10.1016/j.amjopharm.2006.06.002. 16860262

[48] Zheng Kang L, Suministrado MSP, Chen C et al. Medication compliance in Singaporean patients with Alzheimer's disease. Singapore Med J 2019;60:154–60. 10.11622/smedj.2018076. 29931376 PMC6441685

[49] Osada T, Watanabe N, Asano N et al. Adverse drug events affecting medication persistence with rivastigmine patch application. Patient Prefer Adherence 2018;12:1247–52. 10.2147/PPA.S166680. 30050286 PMC6055883

[50] Pariente A, Fourrier-Réglat A, Bazin F et al. Effect of treatment gaps in elderly patients with dementia treated with cholinesterase inhibitors. Neurology 2012;78:957–63. 10.1212/wnl.0b013e31824d5773. 22422894

[51] Amuah JE, Hogan DB, Eliasziw M et al. Persistence with cholinesterase inhibitor therapy in a population-based cohort of patients with Alzheimer's disease. Pharmacoepidemiol Drug Saf 2010;19:670–9. 10.1002/pds.1946. 20583207

[52] Borah B, Sacco P, Zarotsky V. Predictors of adherence among Alzheimer's disease patients receiving oral therapy. Curr Med Res Opin 2010;26:1957–65. 10.1185/03007995.2010.493788. 20569067

[53] Fisher A, Carney G, Bassett K et al. Tolerability of cholinesterase inhibitors: a population-based study of persist-ence, adherence, and switching. Drugs Aging 2017;34:221–31. 10.1007/s40266-017-0438-x. 28138912

[54] Fisher A, Carney G, Bassett K et al. Cholinesterase inhibitor utilization: the impact of provincial drug policy on discontinuation. Value Health 2016;19:688–96. 10.1016/j.jval.2016.03.1832. 27565287

[55] Herrmann N, Binder C, Dalziel W et al. Persistence with cholinesterase inhibitor therapy for dementia: an observational administrative health database study. Drugs Aging 2009;26:403–7. 10.2165/00002512-200926050-00004. 19552492

[56] Herrmann N, Gill SS, Bell CM et al. A population-based study of cholinesterase inhibitor use for dementia. J Am Geriatr Soc 2007;55:1517–23. 10.1111/j.1532-5415.2007.01377.x. 17697100

[57] Kogut SJ, El-Maouche D, Abughosh SM. Decreased persistence to cholinesterase inhibitor therapy with concomitant use of drugs that can impair cognition. Pharmacotherapy 2005;25:1729–35. 10.1592/phco.2005.25.12.1729. 16305292

[58] Kröger E, van Marum R, Souverein P et al. Discontinuation of cholinesterase inhibitor treatment and determinants thereof in the Netherlands: a retrospective cohort study. Drugs Aging 2010;27:663–75. 10.2165/11538230-000000000-00000. 20658794

[59] Le Couteur DG, Robinson M, Leverton A et al. Adherence, persistence and continuation with cholinesterase inhibitors in Alzheimer's disease. Australas J Ageing 2012;31:164–9. 10.1111/j.1741-6612.2011.00564.x. 22950587

[60] Minthon L, Wallin AK, Eriksson S et al. Long-term rivastigmine treatment in a routine clinical setting. Acta Neurol Scand 2009;119:180–5. 10.1111/j.1600-0404.2008.01086.x. 18759798

[61] Nakagawa R, Ohnishi T, Kobayashi H et al. Long-term effect of galantamine on cognitive function in patients with Alzheimer's disease versus a simulated disease trajectory: an observational study in the clinical setting. Neuropsychiatr Dis Treat 2017;13:1115–24. 10.2147/NDT.S133145. 28458553 PMC5402999

[62] Nazir E, Mushtaq M. A prospective study on the use of rivastigmine transdermal patch in Alzheimer's dementia in a routine clinical setting. Dement Neuropsychol 2010;4:245–9. 10.1590/S1980-57642010DN40300014. 29213693 PMC5619296

[63] Olchanski N, Daly AT, Zhu Y et al. Alzheimer's disease medication use and adherence patterns by race and ethnicity. Alzheimers Dement 2023;19:1184–93. 10.1002/alz.12753. 35939325 PMC9905357

[64] Tu Q, Zou Y, Zhang M et al. Application status of memantine in patients with dementia in the Chongqing area of Southwest China. J Clin Gerontol Geriatr 2015;6:85–8. 10.1016/j.jcgg.2015.02.003.

[65] Umegaki H, Itoh A, Suzuki Y et al. Discontinuation of donepezil for the treatment of Alzheimer's disease in geriatric practice. Int Psychogeriatr 2008;20:800–6. 10.1017/S1041610208007011. 18341753

[66] Park KH, Yang Y, Chen C et al. Discontinuation rate of newly prescribed donepezil in Alzheimer's disease patients in Asia. J Clin Neurol 2021;17:376–84. 10.3988/jcn.2021.17.3.376. 34184445 PMC8242303

[67] Ndukwe HC, Nishtala PS. Donepezil adherence, persistence and time to first discontinuation in a three-year follow-up of older people. Dement Geriatr Cogn Disord Extra 2015;5:482–91. 10.1159/000441894. PMC4777950 26955381

[68] Kadohara K, Sato I, Doi Y et al. Prescription patterns of medications for Alzheimer's disease in Japan from 2010 to 2015: a descriptive pharmacy claims database study. Neurol Ther 2017;6:25–37. 10.1007/s40120-016-0057-1. PMC5447550 27896785

[69] Gill SS, Bronskill SE, Mamdani M et al. Representation of patients with dementia in clinical trials of donepezil. Can J Clin Pharmacol 2004;11:e274–85. 15604527

[70] Pariente A, Pinet M, Moride Y et al. Factors associated with persistence of cholinesterase inhibitor treatments in the elderly. Pharmacoepidemiol Drug Saf 2010;19:680–6. 10.1002/pds.1933. 20583209

[71] Thorpe CT, Fowler NR, Harrigan K et al. Racial and ethnic differences in initiation and discontinuation of antidementia drugs by medicare beneficiaries. J Am Geriatr Soc 2016;64:1806–14. 10.1111/jgs.14403. 27549029 PMC5026892

[72] Suh DC, Thomas SK, Valiyeva E et al. Drug persistency of two cholinesterase inhibitors: rivastigmine versus donepezil in elderly patients with Alzheimer's disease. Drugs Aging 2005;22:695–707. 10.2165/00002512-200522080-00006. 16060719

[73] Saleh S, Kirk A, Morgan DG et al. Less education predicts anticholinesterase discontinuation in dementia patients. Can J Neurol Sci 2013;40:684–90. 10.1017/S031716710001492X. 23968942

[74] Abughosh SM, Kogut SJ. Comparison of persistence rates of acetylcholine-esterase inhibitors in a state Medicaid program. Patient Prefer Adherence 2008;2:79–85. 10.2147/ppa.s2652. 19920947 PMC2770391

[75] Rungsanpanya T, Muangpaisan W, Praditsuwan R. Clinical practice with antidementia drugs in a geriatric clinic. J Med Assoc Thai 2012;95:1081–9. 23061314

[76] Seibert J, Tracik F, Articus K et al. Effectiveness and tolerability of transdermal rivastigmine in the treatment of Alzheimer's disease in daily practice. Neuropsychiatr Dis Treat 2012;8:141–7. 10.2147/NDT.S29116. 22536070 PMC3333784

[77] Lai TH, Wang WF, Yip BS et al. Real-world evaluation of compliance and preference in Alzheimer's disease treatment: an observational study in Taiwan. Patient Prefer Adherence 2016;10:383–90. 10.2147/PPA.S95271. 27099476 PMC4821393

[78] Maclagan LC, Bronskill SE, Guan J et al. Predictors of cholinesterase discontinuation during the first year after nursing home admission. J Am Med Dir Assoc 2018;19:959–9. 10.1016/j.jamda.2018.07.020. 30262440

[79] Mador J, Hecker J, Clark M. Evaluation of donepezil in Alzheimer's disease – experience from an Australian memory clinic. Australas J Ageing 2003;22:146–50.

[80] Kim YJ, So KY, Lee HM et al. Changes in dementia treatment patterns associated with changes in the National Policy in South Korea among patients with newly diagnosed Alzheimer's disease between 2011 and 2017: results from the multicenter, retrospective CAPTAIN study. BMC Public Health 2024;24:168. 10.1186/s12889-024-17671-2. 38216922 PMC10787419

[81] Hsieh S-W, Chen J-C, Chen N-C et al. Real-world evaluation of tolerability, safety and efficacy of rivastigmine oral solution in patients with mild to moderate Alzheimer's disease dementia. Clin Psychopharmacol Neurosci 2021;19:459–69. 10.9758/cpn.2021.19.3.459. 34294615 PMC8316665

[82] Mossello E, Tonon E, Caleri V et al. Effectiveness and safety of cholinesterase inhibitors in elderly subjects with Alzheimer's disease: a “real world” study. Arch Gerontol Geriatr 2004;38:297–307. 10.1016/j.archger.2004.04.040. 15207427

[83] Olazaran J, Carnero-Pardo C, Fortea J et al. Prevalence of treated patients with Alzheimer's disease: current trends and COVID-19 impact. Alzheimers Res Ther 2023;15:130. 10.1186/s13195-023-01271-0. 37537656 PMC10401753

[84] Niznik JD, Zhao X, He M et al. Factors associated with deprescribing acetylcholinesterase inhibitors in older nursing home residents with severe dementia. J Am Geriatr Soc 2019;67:1871–9. 10.1111/jgs.15985. 31162642 PMC7456032

[85] Lim EY, Yang DW, Kim JS et al. Safety and efficacy of anti-dementia agents in the extremely elderly patients with dementia. J Korean Med Sci 2018;33:e133. 10.3346/jkms.2018.33.e133. 29736156 PMC5934516

[86] Chang C-C, Peng G-S, Lai T-J et al. A 48-week, multicenter, open-label, observational study evaluating oral rivastigmine in patients with mild-to-moderate Alzheimer's disease in Taiwan. Adv Ther 2019;36:1455–64. 10.1007/s12325-019-00939-0. 30953330

[87] Frankfort SV, Appels BA, de Boer A et al. Discontinuation of rivastigmine in routine clinical practice. Int J Geriatr Psychiatry 2005;20:1167–71. 10.1002/gps.1411. 16315150

[88] Roe CM, Anderson MJ, Spivack B. Brief report: how many patients complete an adequate trial of donepezil? Alzheimer Dis Assoc Disord 2002;16:49–51. 10.1097/00002093-200201000-00008. 11882749

[89] Chang CC, Chan L, Chou HH et al. Effectiveness of the 10 cm^2^ rivastigmine patch in Taiwanese patients with mild-to-moderate Alzheimer's dementia: a 48-week real-world observational study. Adv Ther 2021;38:5286–301. 10.1007/s12325-021-01893-6. 34506009 PMC8478746

[90] Van Der Putt R, Dineen C, Janes D et al. Effectiveness of acetylcholinesterase inhibitors: diagnosis and severity as predictors of response in routine practice. Int J Geriatr Psychiatry 2006;21:755–60. 10.1002/gps.1557. 16906631

[91] Wallin AK, Andreasen N, Eriksson S et al. Donepezil in Alzheimer's disease: what to expect after 3 years of treatment in a routine clinical setting. Dement Geriatr Cogn Disord 2007;23:150–60. 10.1159/000098052. 17312368

[92] Sonde L, Johnell K. Is drug treatment for dementia followed up in primary care? A Swedish study of dementia clinics and referring primary care centres. PloS One 2013;8:e57161. 10.1371/journal.pone.0057161. 23437334 PMC3577769

[93] Cagnin A, Cester A, Costa B et al. Effectiveness of switching to the rivastigmine transdermal patch from oral cholinesterase inhibitors: a naturalistic prospective study in Alzheimer's disease. Neurol Sci 2015;36:457–63. 10.1007/s10072-014-2002-3. 25394739

[94] Matthews HP, Korbey J, Wilkinson DG et al. Donepezil in Alzheimer's disease: eighteen month results from Southampton memory clinic. Int J Geriatr Psychiatry 2000;15:713–20. 10.1002/1099-1166(200008)15:8<713::aid-gps187>3.0.co;2-i. 10960883

[95] Stamouli SS, Tzanakaki M, Giatas S et al. An open-label, multicenter observational study for patients with Alzheimer's disease treated with memantine in the clinical practice. Dement Geriatr Cogn Dis Extra 2011;1:10–9. 10.1159/000322882. 22163229 PMC3199890

[96] Wurm R, Stamm T, Reichardt B et al. Prescription patterns of antidementives in a high income country: a pharmacoepidemiologic study. Alzheimers Dement: Transl Res Clin Interv 2020;6:1–6. 10.1002/trc2.12014. PMC7189407 32355871

[97] Vidal JS, Lacombe JM, Dartigues JF et al. Memantine therapy for Alzheimer disease in real-world practice: an observational study in a large representative sample of French patients. Alzheimer Dis Assoc Disord 2008;22:125–30. 10.1097/WAD.0b013e31815a9e10. 18525283

[98] Chang CJ, Chou TC, Chang CC et al. Persistence and adherence to rivastigmine in patients with dementia: results from a noninterventional, retrospective study using the National Health Insurance research database of Taiwan. Alzheimers Dement 2019;5:46–51. 10.1016/j.trci.2018.06.013. PMC6360604 30766912

[99] Ferreira D, Nogueira N, Guimarães J et al. Anti-dementia drugs: what is the evidence in advanced stages? Porto Biomed J 2024;9:251. 10.1097/j.pbj.0000000000000251. 38690178 PMC11060217

[100] Parsons C, Lim WY, Loy C et al. Withdrawal or continuation of cholinesterase inhibitors or memantine or both, in people with dementia. Cochrane Database Syst Rev 2021;2:Cd009081. 10.1002/14651858.CD009081.pub2. 35608903 PMC8094886

[101] Joe E, Ringman JM. Cognitive symptoms of Alzheimer's disease: clinical management and prevention. BMJ 2019;367:l6217. 10.1136/bmj.l6217. 31810978

[102] Assimon MM . Confounding in observational studies evaluating the safety and effectiveness of medical treatments. Kidney360 2021;2:1156–9. 10.34067/kid.0007022020. 35368357 PMC8786092

[103] Simard P, Presse N, Roy L et al. Persistence and adherence to oral antidiabetics: a population-based cohort study. Acta Diabetol 2015;52:547–56. 10.1007/s00592-014-0692-x. 25524433

[104] Kröger E, Mouls M, Wilchesky M et al. Adverse drug reactions reported with cholinesterase inhibitors: an analysis of 16 years of individual case safety reports from VigiBase. Ann Pharmacother 2015;49:1197–206. 10.1177/1060028015602274. 26324356

[105] Isik AT, Soysal P, Stubbs B et al. Cardiovascular outcomes of cholinesterase inhibitors in individuals with dementia: a meta-analysis and systematic review. J Am Geriatr Soc 2018;66:1805–11. 10.1111/jgs.15415. 29851022

[106] Ruangritchankul S, Chantharit P, Srisuma S et al. Adverse drug reactions of acetylcholinesterase inhibitors in older people living with dementia: a comprehensive literature review. Ther Clin Risk Manag 2021;17:927–49. 10.2147/TCRM.S323387. 34511919 PMC8427072

[107] Jones RW . A review comparing the safety and tolerability of memantine with the acetylcholinesterase inhibitors. Int J Geriatr Psychiatry 2010;25:547–53. 10.1002/gps.2384. 20049770

