# Supplementary material for: Persistence with anti-dementia medications: a systematic review and meta-analysis
Source: Age Ageing. 2025 Jun 3;54(6):afaf151. doi: 10.1093/ageing/afaf151 (PMC12131239; doi:10.1093/ageing/afaf151)
Supplement: aa-24-2572-File002_afaf151 [file aa-24-2572-file002_afaf151.docx]

**Persistence with anti-dementia medications: a systematic review and meta-analysis**

[Appendix A: Search strategy 2](#_Toc182356394)

[Appendix B: The JBI quality assessment scores for included studies 7](#_Toc182356395)

[Appendix C: Datapoints retrieved from included articles 10](#_Toc182356396)

[Appendix D: Funnel plot for the percentage persistent with ChEIs and memantine at 12 months. 13](#_Toc182356397)

[Appendix E: Sensitivity analysis of meta-analysis using leave-one-out method 14](#_Toc182356398)

[Appendix F: Galbraith plot of meta-analysis 15](#_Toc182356399)

[Appendix G: Subgroup analysis: 16](#_Toc182356400)

[Appendix H: Persistent rate with ChEIs and memantine in prospective studies 20](#_Toc182356401)

[Appendix I: Forest-plot for follow up durations of 4-7, 24 and 36 months 21](#_Toc182356402)

[Appendix J. PRISMA guidelines checklist 22](#_Toc182356403)

[Appendix K: PRISMA for abstract checklist 25](#_Toc182356404)

# Appendix A: Search strategy

**Table 1: search strategy and results for Medline**

| No | Concept | Search terms | Results |
| --- | --- | --- | --- |
| 1 | Dementia | exp Dementia/ | 211731 |
| 2 |  | (Alzheimer* or dement* or cognit*).ab,kf,ti. | 746359 |
| 3 |  | 1 OR 2 | 782946 |
| 4 | Antidementia Drugs | exp Cholinesterase Inhibitors/ | 55323 |
| 5 |  | Memantine/ | 2677 |
| 6 |  | (acetylcholinesterase inhibitor* or anticholinesterase agent* or anti cholinesterase* or anticholinesterase drug* or cholinesterase inhibitor* or donepezil or rivastigmine or galantamine or memantine or aducanumab or lecanemab or tacrine).ab,kf,ti,nm. | 35968 |
| 7 |  | (Antidement* or anti-dement* or AntiAlzheimer* or anti-Alzheimer*).ab,kf,ti. | 2609 |
| 8 |  | 4 OR 5 OR 6 OR 7 | 66042 |
| 9 | Medication Persistence | exp "Treatment Adherence and Compliance"/ | 278229 |
| 10 |  | exp Withholding Treatment/ | 16210 |
| 11 |  | (noncomplian* or non-complian* or adheren* or nonadheren* or non-adheren* or persisten* or nonpersisten* or non-persisten* or withholding Treatment* or cessation or ceas* or medication duration or medication continuation or discontinuation).ab,kf,ti. | 792399 |
| 12 |  | ((complian* or noncomplian* or non-complian* or adheren* or nonadheren* or non-adheren* or persisten* or nonpersisten* or non-persisten* or cooperat* or co-operat* or concordan* refusal or withdrawal or discontinu* cessation or ceas* or interrupt* or dropout or drop-out) adj6 (patient* or treatment* or medication* or Therap* or regimen)).ab,kf,ti. | 245297 |
| 13 |  | 9 OR 10 OR 11 OR 12 | 1104892 |
| 14 |  | 3 AND 9 AND 13 | 865 |
| 15 |  | limit 15 to yr="1995 -Current" | 846 |
| 16 |  | 16 NOT exp animals/ not humans.sh. | 752 |

**Table 2: search strategy and results for EMBASE (MeSH terms were find via Emtree)**

| No | Concept | Search terms | Results |
| --- | --- | --- | --- |
| 1 | Dementia | exp dementia/ | 456143 |
| 2 |  | (Alzheimer* or dement* or cognit*).ab,kf,ti. | 1013604 |
| 3 |  | 1 OR 2 | 1129185 |
| 4 | Antidementia Drugs | exp cholinesterase inhibitor/ | 96095 |
| 5 |  | Memantine/ | 13234 |
| 6 |  | ("acetylcholinesterase inhibitor*" or "anticholinesterase agent*" or "anti cholinesterase*" or "anticholinesterase drug*" or "cholinesterase inhibitor*" or donepezil or rivastigmine or galantamine or memantine or aducanumab or lecanemab or tacrine).ab,kf,ti. | 28705 |
| 7 |  | (Antidement* or anti-dement* or AntiAlzheimer* or anti-Alzheimer*).ab,kf,ti. | 3723 |
| 8 |  | 4 OR 5 OR 6 OR 7 | 108569 |
| 9 | Medication Persistence | exp patient compliance/ | 196257 |
| 10 |  | exp treatment withdrawal/ | 283463 |
| 11 |  | (noncomplian* or non-complian* or adheren* or nonadheren* or non-adheren* or persisten* or nonpersisten* or non-persisten* or "withholding Treatment*" or cessation or ceas* or "medication duration" or "medication continuation" or discontinuation).ab,kf,ti. | 1141882 |
| 12 |  | ((complian* or noncomplian* or non-complian* or adheren* or nonadheren* or non-adheren* or persisten* or nonpersisten* or non-persisten* or cooperat* or co-operat* or "concordan* refusal" or withdrawal or "discontinu* cessation" or ceas* or interrupt* or dropout or drop-out) adj6 (patient* or treatment* or medication* or Therap* or regimen)).ab,kf,ti. | 407372 |
| 13 |  | 9 OR 10 OR 11 OR 12 | 1565738 |
| 14 |  | 3 AND 8 AND 13 | 3084 |
| 15 |  | limit 14 to yr="1995 -Current" | 3059 |
| 16 |  | 15 NOT exp animal experiment/ | 2912 |

**Table 3: search strategy and results for PsycINFO (MeSH terms were find via PsycINFO Thesaurus (**[**PsycInfo via Ovid**](http://ezproxy.utas.edu.au/login?url=http://ovidsp.ovid.com/ovidweb.cgi?T=JS&NEWS=n&CSC=Y&PAGE=main&D=psyh)**))**

| No | Concept | Search terms | Results |
| --- | --- | --- | --- |
| 1 | Dementia | exp dementia/ | 94255 |
| 2 |  | (Alzheimer* or dement* or cognit*).ab,id,ti. | 643810 |
| 3 |  | 1 OR 2 | 644583 |
| 4 | Antidementia Drugs | exp cholinesterase inhibitor/ | 2628 |
| 5 |  | ("acetylcholinesterase inhibitor*" or "anticholinesterase agent*" or "anti cholinesterase*" or "anticholinesterase drug*" or "cholinesterase inhibitor*" or donepezil or rivastigmine or galantamine or memantine or aducanumab or lecanemab or tacrine).ab,id,ti. | 5776 |
| 6 |  | (Antidement* or anti-dement* or AntiAlzheimer* or anti-Alzheimer*).ab,id,ti. | 616 |
| 7 |  | 4 OR 5 OR 6 | 6903 |
| 8 | Medication Persistence | exp Treatment Compliance/ | 18071 |
| 9 |  | exp Therapeutic Processes/ | 84235 |
| 10 |  | exp Drug Withdrawal/ | 9942 |
| 11 |  | exp Treatment Withholding/ | 1133 |
| 12 |  | ("Treatment Compliance" or "Therapeutic Processes" or noncomplian* or non-complian* or adheren* or nonadheren* or non-adheren* or persisten* or nonpersisten* or non-persisten* or "withholding Treatment*" or cessation or ceas* or "medication duration" or "medication continuation" or discontinuation or "Drug Withdrawal" or "treatment withholding").ab,id,ti. | 152557 |
| 13 |  | ((complian* or noncomplian* or non-complian* or adheren* or nonadheren* or non-adheren* or persisten* or nonpersisten* or non-persisten* or cooperat* or co-operat* or "concordan* refusal" or withdrawal or "discontinu* cessation" or ceas* or interrupt* or dropout or drop-out) adj6 (patient* or treatment* or medication* or Therap* or regimen)).ab,id,ti. | 47157 |
| 14 |  | 8 OR 9 OR 10 OR 11 OR 12 OR 13 | 255018 |
| 15 |  | 3 AND 7 AND 14 | 408 |
| 16 |  | limit 15 to yr="1995 -Current" | 405 |
| 17 |  | 16 NOT exp animal research/ | 405 |

**Table 4: search strategy and results for CINAHL**

| No | Concept | Search terms | Results |
| --- | --- | --- | --- |
| 1 | Dementia | (MH "Dementia+") | 85282 |
| 2 |  | TI ( Alzheimer* OR dement* OR cognit* ) OR AB ( Alzheimer* OR dement* OR cognit* ) OR SU ( Alzheimer* OR dement* OR cognit* ) | 308857 |
| 3 |  | S1 OR S2 | 310424 |
| 4 | Antidementia Drugs | (MH "Cholinesterase Inhibitors+") OR (MH "Memantine") | 5220 |
| 5 |  | (TI "acetylcholinesterase inhibitor*" OR AB "acetylcholinesterase inhibitor*" OR SU "acetylcholinesterase inhibitor*") OR (TI "anticholinesterase agent*" OR AB "anticholinesterase agent*" OR SU "anticholinesterase agent*") OR (TI "anti cholinesterase*" OR AB "anti cholinesterase*" OR SU "anti cholinesterase*") OR (TI "anticholinesterase drug*" OR AB "anticholinesterase drug*" OR SU "anticholinesterase drug*") OR (TI "cholinesterase inhibitor*" OR AB "cholinesterase inhibitor*" OR SU "cholinesterase inhibitor*") OR (TI donepezil OR AB donepezil OR SU donepezil) OR (TI rivastigmine OR AB rivastigmine OR SU rivastigmine) OR (TI galantamine OR AB galantamine OR SU galantamine) OR (TI memantine OR AB memantine OR SU memantine) OR (TI aducanumab OR AB aducanumab OR SU aducanumab) OR (TI lecanemab OR AB lecanemab OR SU lecanemab) OR (TI tacrine OR AB tacrine OR SU tacrine) | 6300 |
| 6 |  | ((TI Antidement* OR AB Antidement* OR SU Antidement*) OR (TI anti-dement* OR AB anti-dement* OR SU anti-dement*) OR (TI AntiAlzheimer* OR AB AntiAlzheimer* OR SU AntiAlzheimer*) OR (TI anti-Alzheimer* OR AB anti-Alzheimer* OR SU anti-Alzheimer*)) | 431 |
| 7 |  | S4 OR S5 OR S6 | 7184 |
| 8 | Medication Persistence | (MH "Medication Compliance") OR (MH "Treatment Duration") OR (MH "Treatment Withdrawal") OR (MH "Compliance with Medical Regimen (Saba CCC)") OR MH "Treatment Interruption") | 36714 |
| 9 |  | ((TI noncomplian* OR AB noncomplian* OR SU noncomplian*) OR (TI non-complian* OR AB non-complian* OR SU non-complian*) OR (TI adheren* OR AB adheren* OR SU adheren*) OR (TI nonadheren* OR AB nonadheren* OR SU nonadheren*) OR (TI non-adheren* OR AB non-adheren* OR SU non-adheren*) OR (TI persisten* OR AB persisten* OR SU persisten*) OR (TI nonpersisten* OR AB nonpersisten* OR SU nonpersisten*) OR (TI non-persisten* OR AB non-persisten* OR SU non-persisten*) OR (TI "withholding Treatment*" OR AB "withholding Treatment*" OR SU "withholding Treatment*") OR (TI cessation OR AB cessation OR SU cessation) OR (TI ceas* OR AB ceas* OR SU ceas*) OR (TI "medication duration" OR AB "medication duration" OR SU "medication duration") OR (TI "medication continuation" OR AB "medication continuation" OR SU "medication continuation") OR (TI discontinuation OR AB discontinuation OR SU discontinuation)) | 214204 |
| 10 |  | (((TI complian* OR AB complian* OR SU complian*) OR (TI noncomplian* OR AB noncomplian* OR SU noncomplian*) OR (TI non-complian* OR AB non-complian* OR SU non-complian*) OR (TI adheren* OR AB adheren* OR SU adheren*) OR (TI nonadheren* OR AB nonadheren* OR SU nonadheren*) OR (TI non-adheren* OR AB non-adheren* OR SU non-adheren*) OR (TI persisten* OR AB persisten* OR SU persisten*) OR (TI nonpersisten* OR AB nonpersisten* OR SU nonpersisten*) OR (TI non-persisten* OR AB non-persisten* OR SU non-persisten*) OR (TI cooperat* OR AB cooperat* OR SU cooperat*) OR (TI co-operat* OR AB co-operat* OR SU co-operat*) OR (TI "concordan* refusal" OR AB "concordan* refusal" OR SU "concordan* refusal") OR (TI withdrawal OR AB withdrawal OR SU withdrawal) OR (TI "discontinu* cessation" OR AB "discontinu* cessation" OR SU "discontinu* cessation") OR (TI ceas* OR AB ceas* OR SU ceas*) OR (TI interrupt* OR AB interrupt* OR SU interrupt*) OR (TI dropout OR AB dropout OR SU dropout) OR (TI drop-out OR AB drop-out OR SU drop-out)) N6 ((TI patient* OR AB patient* OR SU patient*) OR (TI treatment* OR AB treatment* OR SU treatment*) OR (TI medication* OR AB medication* OR SU medication*) OR (TI Therap* OR AB Therap* OR SU Therap*) OR (TI regimen OR AB regimen OR SU regimen))) | 119808 |
| 11 |  | S8 OR S9 OR S10 | 279880 |
| 12 |  | S3 AND S7 AND S11 | 363 |
| 13 |  | S12 Limiters - Publication Date: 19950101-20240231 | 363 |
| 14 |  | S13 NOT ((MH animals+) NOT (MH humans)) | 359 |

# Appendix B: The JBI quality assessment scores for included studies

| **No** | **Author, year, and country** | **Was the sample frame appropriate to address the target population?** | **Were study participants sampled in an appropriate way?** | **Was the sample size adequate?** | **Were the study subjects and the setting described in detail?** | **Was the data analysis conducted with sufficient coverage of the identified sample?** | **Were valid methods used for the identification of the condition?** | **Was the condition measured in a standard, reliable way for all participants?** | **Was there appropriate statistical analysis?** | **Was the response rate adequate, and if not, was the low response rate managed appropriately?** | **Score** | **Study quality** |
| --- | --- | --- | --- | --- | --- | --- | --- | --- | --- | --- | --- | --- |
| 1 | Ahn et al., 2015, Korea | yes | yes | yes | yes | yes | yes | yes | yes | no | 8 | high |
| 2 | Balazs et al., 2022, Hungary | yes | yes | yes | yes | yes | yes | yes | yes | yes | 9 | high |
| 3 | Bent-Ennakhil et al., 2017, USA | yes | yes | yes | yes | yes | yes | no | yes | yes | 8 | high |
| 4 | Bohlken et al., 2015, Germany | yes | yes | yes | yes | yes | no | yes | yes | yes | 8 | high |
| 5 | Bohlken et al., 2017, Germany | yes | yes | yes | yes | yes | unclear | yes | yes | yes | 8 | high |
| 6 | Byun et al., 2022, Korea | yes | yes | yes | yes | yes | yes | no | yes | yes | 8 | high |
| 7 | Clerici et al., 2009, Italy | no | yes | yes | yes | no | yes | yes | yes | yes | 7 | high |
| 8 | Dybicz et al., 2006, USA | yes | yes | yes | yes | yes | yes | yes | yes | yes | 9 | high |
| 9 | Zheng Kang et al. 2019, Singapore | no | yes | yes | yes | yes | no | yes | yes | no | 6 | moderate |
| 10 | Osada et al., 2018, Japan | no | yes | yes | yes | yes | yes | no | yes | NA | 6 | moderate |
| 11 | Pariente et al. 2012, Canada | yes | yes | yes | yes | yes | yes | yes | yes | yes | 9 | high |
| 12 | Taipale et al., 2014, Finland | yes | yes | yes | yes | yes | yes | no | yes | yes | 8 | high |
| 13 | Amuah, et al., 2010, Canada | yes | yes | yes | yes | yes | yes | yes | yes | yes | 9 | high |
| 14 | Borah et al., 2010, USA | yes | yes | yes | yes | yes | yes | yes | yes | yes | 9 | high |
| 15 | Brewer et al., 2013, Ireland | yes | yes | yes | yes | yes | yes | yes | yes | yes | 9 | high |
| 16 | Fisher et al., 2017, Canada | yes | yes | yes | yes | yes | yes | yes | yes | yes | 9 | high |
| 17 | Fisher et al., 2016, Canada | yes | yes | yes | yes | yes | yes | yes | yes | yes | 9 | high |
| 18 | Fukuda et al., 2022, Japan | yes | yes | yes | yes | yes | no | yes | yes | yes | 8 | high |
| 19 | Gardette et al., 2014, 12 European countries | yes | yes | yes | yes | yes | yes | yes | yes | yes | 9 | high |
| 20 | Haider et al., 2014, Austria | yes | yes | yes | yes | yes | no | yes | yes | yes | 8 | high |
| 21 | Herrmann et al., 2009, Canada | yes | yes | yes | yes | yes | no | no | yes | yes | 7 | high |
| 22 | Hermann et al., 2007, Canada | yes | yes | yes | yes | yes | no | no | no | yes | 6 | moderate |
| 23 | Kogut et al, 2005, USA | yes | yes | yes | yes | yes | yes | yes | yes | NA | 8 | high |
| 24 | Kongpakwattana et al., 2019 Thailand | yes | yes | yes | yes | yes | no | yes | yes | yes | 8 | high |
| 25 | Kröger et al., 2010, Netherlands | yes | yes | yes | yes | yes | yes | yes | yes | yes | 9 | high |
| 26 | Ku et al., 2018, Taiwan | yes | yes | yes | yes | yes | no | yes | yes | yes | 8 | high |
| 27 | Le Couteur et al, 2012, Australia | yes | yes | yes | yes | yes | yes | yes | yes | yes | 9 | high |
| 28 | Minthon et al, 2009, Sweden | yes | unclear | yes | yes | yes | yes | yes | yes | yes | 8 | high |
| 29 | Nakagawa et al., 2017, Japan | yes | unclear | yes | yes | yes | no | yes | yes | yes | 7 | high |
| 30 | Nazir et al., 2010, UK | no | no | no | yes | yes | yes | yes | yes | yes | 6 | moderate |
| 31 | Olchanski, 2023, USA | yes | yes | yes | yes | yes | no | yes | yes | yes | 8 | high |
| 32 | Tu et al., 2015, China | no | yes | no | yes | yes | yes | no | yes | no | 5 | moderate |
| 33 | Umegaki et al., 2008, Japan | no | unclear | yes | yes | yes | no | no | yes | NA | 4 | moderate |
| 34 | Park et al., 2021, Asia | yes | unclear | yes | yes | yes | yes | yes | yes | yes | 8 | high |
| 35 | Ndukwe et al., 2015, New Zealand | yes | yes | yes | yes | yes | yes | yes | yes | yes | 9 | high |
| 36 | Kadohara et al., 2017, Japan | yes | yes | yes | yes | yes | yes | yes | yes | yes | 9 | high |
| 37 | Gill et al., 2004, Canada | yes | yes | yes | yes | yes | yes | yes | yes | yes | 9 | high |
| 38 | Olazaran et al., 2013, Spain | yes | unclear | yes | yes | yes | yes | yes | yes | yes | 8 | high |
| 39 | Pariente et al., 2010, France | yes | yes | yes | yes | yes | no | yes | yes | yes | 8 | high |
| 40 | Thorpe et al., 2016, USA | yes | yes | yes | yes | yes | yes | yes | yes | NA | 8 | high |
| 41 | Suh et al., 2005, USA | yes | yes | yes | yes | yes | yes | yes | yes | NA | 8 | high |
| 42 | Steininger et al., 2020, Germany and UK | yes | yes | yes | yes | yes | no | yes | yes | NA | 7 | high |
| 43 | Saleh et al., 2013, Canada | no | yes | no | yes | yes | yes | yes | yes | yes | 7 | high |
| 44 | Abughosh et al., 2008, USA | yes | yes | yes | yes | yes | no | yes | yes | no | 7 | high |
| 45 | Rungsanpanya et al., 2012, Thailand | no | unclear | no | yes | yes | yes | no | yes | yes | 5 | moderate |
| 46 | Seibert et al., 2012, Germany | yes | unclear | yes | no | yes | yes | yes | yes | unclear | 6 | moderate |
| 47 | Lai et al., 2016, Taiwan | yes | yes | yes | yes | yes | yes | yes | yes | yes | 9 | high |
| 48 | Maclagan et al., 2018, Canada | yes | yes | yes | yes | yes | yes | yes | yes | yes | 9 | high |
| 49 | Mador et al., 2003, Australia | no | yes | no | no | unclear | yes | no | no | yes | 3 | low |
| 50 | Kim et al., 2024, South Korea | yes | yes | yes | yes | unclear | yes | no | no | yes | 6 | moderate |
| 51 | Hsieh et al., 2021, Taiwan | yes | yes | yes | yes | no | yes | yes | yes | unclear | 7 | high |
| 52 | Mossello et al., 2004, Italy | yes | yes | yes | yes | no | yes | yes | yes | yes | 8 | high |
| 53 | Olazaran et al., 2023, Spain | yes | yes | yes | yes | yes | no | no | yes | yes | 7 | high |
| 54 | Niznik et al., 2019, | yes | yes | yes | yes | yes | yes | yes | yes | yes | 9 | high |
| 55 | Lim et al., 2018, Korea | no | unclear | yes | no | no | no | no | yes | unclear | 2 | low |
| 56 | Chang, 2019, Taiwan | yes | unclear | yes | no | no | yes | yes | yes | unclear | 5 | moderate |
| 57 | Frankfort et al., 2005, Netherland | no | no | yes | yes | no | yes | no | yes | unclear | 4 | moderate |
| 58 | Roe et al., 2002, US | yes | yes | no | yes | no | yes | yes | yes | yes | 7 | high |
| 59 | Chang et al., 2021, Taiwan | yes | unclear | yes | no | unclear | yes | yes | yes | unclear | 5 | moderate |
| 60 | Van Der Putt et al., 2006, UK | yes | unclear | yes | no | unclear | yes | yes | yes | yes | 6 | moderate |
| 61 | Wallin et al., 2007, Sweden | yes | unclear | yes | no | unclear | yes | yes | yes | yes | 6 | moderate |
| 62 | Sonde et al., 2013, Sweden | yes | yes | yes | yes | unclear | yes | no | yes | no | 6 | moderate |
| 63 | Cagnin et al, 2015, Italy | yes | unclear | yes | no | yes | yes | yes | yes | yes | 7 | high |
| 64 | Matthews et al., 2000, UK | no | yes | no | no | yes | yes | yes | yes | yes | 6 | moderate |
| 65 | Stamouli et al., 2011, Greece | yes | unclear | yes | yes | yes | yes | yes | yes | yes | 8 | high |
| 66 | Wurm et al., 2020, Austria | yes | yes | yes | yes | yes | yes | no | no | yes | 7 | high |
| 67 | Vidal et al., 2008, France | yes | yes | yes | yes | yes | yes | no | yes | yes | 8 | high |
| 68 | Kostev et al., 2019, Poland | yes | yes | yes | yes | yes | no | yes | yes | yes | 8 | high |

NA: Not Applicable

# Appendix C: Datapoints retrieved from included articles

| **No** | **Study ID (year)** | **Medications** | **Follow-up (months)** | **Permissible gap (days)** | **Persistent** | **Total** | **Standardised*** | **Deceased** | **Lost to follow-up*** | **Switchers** | **Combination therapy** |
| --- | --- | --- | --- | --- | --- | --- | --- | --- | --- | --- | --- |
| 1 | Ahn (2015) | ChEIs | 12 | 30 | 1551 | 6461 | No | 2 | 2 | 3 | 0 |
| 2 | Balazs-A (2022) | donepezil, rivastigmine | 12 | 30 | 2098 | 8433 | No | 2 | 2 | 3 | 0 |
| 3 | Balazs-B (2022) | donepezil, rivastigmine | 24 | 30 | 755 | 8433 | No | 2 | 2 | 3 | 0 |
| 4 | Bohlken-A (2015) | ADD | 12 | 89 | 7746 | 12910 | No | 2 | 2 | 2 | 0 |
| 5 | Bohlken-B (2015) | ChEIs | 12 | 89 | 4945 | 8282 | No | 2 | 2 | 2 | 0 |
| 6 | Bohlken-C (2015) | ChEIs | 36 | 89 | 3627 | 8282 | No | 2 | 2 | 2 | 0 |
| 7 | Bohlken-D (2015) | memantine | 12 | 89 | 2800 | 4628 | No | 2 | 2 | 2 | 0 |
| 8 | Bohlken-E (2015) | memantine | 36 | 89 | 2050 | 4628 | No | 2 | 2 | 2 | 0 |
| 9 | Bohlken-A (2017) | ChEIs | 12 | 180 | 8814 | 11087 | No | 2 | 2 | 2 | 0 |
| 10 | Bohlken-B (2017) | memantine | 12 | 180 | 3914 | 4416 | No | 2 | 2 | 2 | 0 |
| 11 | Byun-A (2022) | ADD | 12 | NA | 3315 | 7054 | No | 2 | 2 | 2 | 3 |
| 12 | Byun-B (2022) | ChEIs | 12 | NA | 2535 | 5489 | No | 2 | 2 | 2 | 3 |
| 13 | Byun-C (2022) | memantine | 12 | NA | 780 | 1565 | No | 2 | 2 | 2 | 3 |
| 14 | Clerici (2009) | memantine | 6 | NA | 264 | 317 | No | 1 | 1 | 1 | 0 |
| 15 | Dybicz (2006) | ChEIs | 12 | 29 | 1601 | 2873 | No | 1 | 1 | 3 | 0 |
| 16 | Lum (2019) | ADD | 12 | 60 | 107 | 135 | Yes | 1 | 3 | 0 | 1 |
| 17 | Osada (2018) | rivastigmine patch | 5.5 | NA | 194 | 312 | No | 1 | 1 | NA | 0 |
| 18 | Pariente (2012) | ChEIs | 12 | 42 | 8219 | 24394 | No | 2 | 2 | 3 | 0 |
| 19 | Taipale-A (2014) | ChEIs | 12 | NA | 4130 | 5162 | No | 1 | 1 | 1 | 3 |
| 20 | Taipale-B (2014) | memantine | 12 | NA | 2049 | 2334 | No | 1 | 1 | 1 | 3 |
| 21 | Amuah-A (2010) | ChEIs | 6.5 | 60 | 540 | 1080 | No | 1 | 2 | 3 | 1 |
| 22 | Amuah-B (2010) | ChEIs | 12 | 60 | 363 | 1080 | No | 1 | 2 | 3 | 1 |
| 23 | Borah (2010) | ADD | 12 | 60 | 1854 | 3091 | No | 1 | 1 | 1 | 1 |
| 24 | Brewer (2013) | ADD | 12 | 63 | 7979 | 14197 | No | 1 | 1 | 1 | 3 |
| 25 | Fisher (2017) | ChEIs | 12 | 30 | 9810 | 22815 | No | 1 | 1 | 3 | 1 |
| 26 | Fisher (2016) | ChEIs | 12 | 90 | 12285 | 25071 | No | 1 | 1 | 2 | 1 |
| 27 | Fukuda-A (2022) | ADD | 12 | 60 | 13206 | 20474 | No | 1 | 0 | 3 | 0 |
| 28 | Fukuda-B (2022) | ADD | 18 | 60 | 11936 | 20474 | No | 1 | 0 | 3 | 0 |
| 29 | Gardette (2014) | ChEIs | 24 | 35 | 164 | 315 | Yes | 1 | 1 | 2 | 0 |
| 30 | Haider (2014) | ADD | 12 | 90 | 6561 | 15809 | No | 1 | 1 | 2 | NA |
| 31 | Herrmann (2009) | ChEIs | 12 | 30 | 2607 | 5622 | No | 0 | 0 | 0 | 0 |
| 32 | Kogut (2005) | ChEIs | 6 | 30 | 872 | 1183 | No | 1 | 1 | 1 | 0 |
| 33 | Kongpakwattana-A (2019) | ADD | 12 | 30 | 147 | 698 | No | 0 | 0 | 3 | 3 |
| 34 | Kongpakwattana-B (2019) | ChEIs | 12 | 30 | 112 | 529 | No | 0 | 0 | 3 | 3 |
| 35 | Kongpakwattana-C (2019) | memantine | 12 | 30 | 35 | 169 | No | 0 | 0 | 3 | 3 |
| 36 | Kröger-A (2010) | ChEIs | 6 | 30 | 2331 | 3369 | No | 1 | 1 | 3 | 0 |
| 37 | Kröger-B (2010) | ChEIs | 36 | 30 | 1381 | 3369 | No | 1 | 1 | 3 | 0 |
| 38 | Ku-A (2018) | ChEIs | 12 | 90 | 5168 | 8614 | No | 1 | 0 | 3 | 0 |
| 39 | Ku-B (2018) | ChEIs | 24 | 90 | 3661 | 8614 | No | 1 | 0 | 3 | 0 |
| 40 | Le Couteur-A (2012) | ChEIs | 12 | 99 | 10173 | 18598 | No | 2 | 2 | 3 | 0 |
| 41 | Le Couteur-B (2012) | ChEIs | 36 | 99 | 6119 | 18598 | No | 2 | 2 | 3 | 0 |
| 42 | Minthon (2009) | rivastigmine | 24 | NA | 135 | 185 | Yes | 1 | 1 | 1 | 1 |
| 43 | Nakagawa-A (2017) | galantamine | 12 | NA | 397 | 661 | No | 0 | 2 | NA | 3 |
| 44 | Nakagawa-B (2017) | galantamine | 18 | NA | 352 | 661 | No | 0 | 2 | NA | 3 |
| 45 | Nazir (2010) | rivastigmine patch | 6 | NA | 24 | 27 | Yes | 1 | 1 | NA | 1 |
| 46 | Olchanski (2023) | ADD | 12 | 45 | 1026 | 1343 | No | 0 | 0 | 3 | 3 |
| 47 | Tu (2015) | memantine | 6 | NA | 41 | 88 | No | 1 | 1 | NA | 3 |
| 48 | Park (2021) | donepezil | 12 | NA | 315 | 385 | Yes | 1 | 1 | 3 | 0 |
| 49 | Ndukwe-A (2015) | donepezil | 12 | 31 | 1019 | 1999 | No | 2 | 2 | 1 | 0 |
| 50 | Ndukwe-B (2015) | donepezil | 36 | 31 | 340 | 1999 | No | 2 | 2 | 1 | 0 |
| 51 | Kadohara (2017) | ChEIs | 12 | 60 | 60887 | 103592 | No | 2 | 2 | 0 | 3 |
| 52 | Gill (2004) | donepezil | 7 | 180 | 4638 | 6424 | No | 2 | 2 | 1 | 0 |
| 53 | Olazaran-A (2013) | ChEIs | 36 | NA | 150 | 194 | No | 1 | 1 | 3 | 0 |
| 54 | Olazaran -B (2013) | ChEIs | 12 | NA | 188 | 240 | No | 1 | 1 | 3 | 0 |
| 55 | Pariente (2010) | ChEIs | 12 | 60 | 427 | 942 | No | 0 | 0 | 0 | 0 |
| 56 | Thorpe-A (2016) | ADD | 12 | 30 | 1396 | 3481 | No | 1 | 1 | 3 | 3 |
| 57 | Thorpe-B (2016) | ChEIs | 12 | 30 | 952 | 2390 | No | 1 | 1 | 3 | 3 |
| 58 | Thorpe-C (2016) | memantine | 12 | 30 | 376 | 921 | No | 1 | 1 | 3 | 3 |
| 59 | Suh (2005) | ChEIs | 12 | 30 | 395 | 783 | No | 1 | 1 | 3 | 3 |
| 60 | Steininger-A (2020) | ADD | 12 | 90 | 2132 | 3863 | No | 0 | 0 | 0 | 0 |
| 61 | Steininger-B (2020) | ADD | 12 | 90 | 2680 | 3342 | No | 0 | 0 | 0 | 0 |
| 62 | Saleh (2013) | ChEIs | 6 | NA | 44 | 63 | No | 1 | 1 | 3 | 0 |
| 63 | Abughosh-A (2008) | ChEIs | 12 | 180 | 686 | 1199 | No | 0 | 0 | 3 | 0 |
| 64 | Abughosh-B (2008) | ChEIs | 24 | 180 | 124 | 816 | No | 0 | 0 | 3 | 0 |
| 65 | Rungsanpanya-A (2012) | ADD | 12 | NA | 80 | 96 | No | 1 | 1 | 0 | 0 |
| 66 | Rungsanpanya-B (2012) | ChEIs | 12 | NA | 68 | 92 | No | 1 | 1 | 0 | 0 |
| 67 | Rungsanpanya-C (2012) | memantine | 12 | NA | 14 | 16 | No | 1 | 1 | 0 | 0 |
| 68 | Seibert (2012) | rivastigmine patch | 4 | NA | 869 | 1033 | Yes | 1 | 1 | 1 | 0 |
| 69 | Lai (2016) | ADD | 6 | NA | 236 | 270 | Yes | 1 | 1 | 3 | NA |
| 70 | Maclaga (2018) | ChEIs | 12 | 30 | 3110 | 13416 | No | 1 | 1 | 3 | 0 |
| 71 | Hsieh (2021) | rivastigmine oral solution | 12 | NA | 87 | 106 | Yes | 1 | 1 | 2 | NA |
| 72 | Mossello (2004) | ChEIs | 9 | NA | 212 | 407 | Yes | 2 | 2 | 2 | 0 |
| 73 | Olazaran-A (2023) | ADD | 36 | NA | 1566 | 3472 | No | 0 | 0 | 1 | 1 |
| 74 | Olazaran-B (2023) | ChEIs | 36 | NA | 1289 | 2708 | No | 0 | 0 | 1 | 1 |
| 75 | Lim (2018) | ADD | 24 | NA | 121 | 155 | No | 0 | 0 | 2 | 0 |
| 76 | Chang (2019) | rivastigmine | 12 | 14 | 91 | 122 | Yes | 1 | 1 | 1 | 1 |
| 77 | Frankfort-A (2005) | rivastigmine | 12 | NA | 52 | 130 | Yes | 1 | 1 | NA | NA |
| 78 | Frankfort-B (2005) | rivastigmine | 36 | NA | 3 | 116 | Yes | 1 | 1 | NA | NA |
| 79 | Roe (2002) | donepezil | 6 | 14 | 36 | 59 | No | 1 | 1 | NA | NA |
| 80 | Chang (2021) | rivastigmine patch | 11 | NA | 175 | 244 | Yes | 1 | 1 | 3 | 1 |
| 81 | Van Der Putt (2006) | ChEIs | 4 | NA | 939 | 1232 | Yes | 1 | 1 | 0 | 0 |
| 82 | Wallin-A (2007) | donepezil | 12 | NR | 355 | 399 | Yes | 1 | 1 | 1 | 1 |
| 83 | Wallin-B (2007) | donepezil | 36 | NR | 166 | 293 | Yes | 1 | 1 | 1 | 1 |
| 84 | Sonde (2013) | ADD | 24 | NA | 65 | 78 | Yes | 1 | 1 | 0 | 1 |
| 85 | Cagnin (2015) | rivastigmine patch | 6 | NA | 139 | 174 | No | 1 | 2 | 1 | 1 |
| 86 | Matthews-A (2000) | donepezil | 12 | NA | 29 | 80 | No | 1 | 1 | 1 | 1 |
| 87 | Matthews-B (2000) | donepezil | 18 | NA | 12 | 80 | No | 1 | 1 | 1 | 1 |
| 88 | Stamouli (2011) | memantine | 6 | NA | 1680 | 2167 | Yes | 1 | 1 | NA | 3 |
| 89 | Vidal (2008) | memantine | 12 | NA | 2085 | 3258 | No | 2 | 2 | 2 | 3 |
| 90 | Kostev-A (2019) | ChEIs | 12 | 90 | 16515 | 38286 | No | 0 | 0 | 0 | 0 |
| 91 | Kostev-B (2019) | memantine | 12 | 90 | 18283 | 27744 | No | 0 | 0 | 0 | 0 |

Coding for Deceased, Lost to follow-up, Switchers and Combination therapy columns in the scope of persistence: 0= No clarification 1= Excluded (do not counted or no patient received combination therapy), 2= Considered as non-persistent, 3= Considered as persistent

* Where data were available, subjects who died or were lost to follow-up during the study period were excluded from the persistence calculations. Switchers and patients who received combination therapy were considered persistent if the relevant data were provided.

# Appendix D: Funnel plot for the percentage persistent with ChEIs and memantine at 12 months (P = 0.241 at Egger’s test and P = 0.767 and Begg’s test).

# Appendix E: Sensitivity analysis of meta-analysis using leave-one-out method

# Appendix F: Galbraith plot of meta-analysis

# Appendix G: Subgroup analysis:

Figure 1: Subgroup analysis based on permissible gap: equal or less than 45 days, more than 45 days and not required (NR)

Figure 2: Subgroup analysis based on drug groups: ChEIs and memantine

Figure 3: Subgroup analysis based on publication year

Figure 4: Subgroup analysis based on country region: Asia, Europe, North America

# **Appendix H: 12-month persistence rate with ChEIs and memantine in prospective studies**

NR: Not required, ChEIs: Cholinesterase Inhibitors, ADD: Anti-dementia drugs

# Appendix I: Forest-plot for follow up durations of 4-7, 24 and 36 months

ChEIs: Cholinesterase Inhibitors

# Appendix J. PRISMA guidelines checklist

| **Section and Topic** | **Item #** | **Checklist item** | **Location where item is reported** |
| --- | --- | --- | --- |
| **TITLE** | | |  |
| Title | 1 | Identify the report as a systematic review. | Page 1 |
| **ABSTRACT** | | |  |
| Abstract | 2 | See the PRISMA 2020 for Abstracts checklist. | Page 1 |
| **INTRODUCTION** | | |  |
| Rationale | 3 | Describe the rationale for the review in the context of existing knowledge. | Page 2 |
| Objectives | 4 | Provide an explicit statement of the objective(s) or question(s) the review addresses. | Page 2 |
| **METHODS** | | |  |
| Eligibility criteria | 5 | Specify the inclusion and exclusion criteria for the review and how studies were grouped for the syntheses. | Page 2 |
| Information sources | 6 | Specify all databases, registers, websites, organisations, reference lists and other sources searched or consulted to identify studies. Specify the date when each source was last searched or consulted. | Page 2 |
| Search strategy | 7 | Present the full search strategies for all databases, registers and websites, including any filters and limits used. | Page 2 |
| Selection process | 8 | Specify the methods used to decide whether a study met the inclusion criteria of the review, including how many reviewers screened each record and each report retrieved, whether they worked independently, and if applicable, details of automation tools used in the process. | Page 3 |
| Data collection process | 9 | Specify the methods used to collect data from reports, including how many reviewers collected data from each report, whether they worked independently, any processes for obtaining or confirming data from study investigators, and if applicable, details of automation tools used in the process. | Page 3 |
| Data items | 10a | List and define all outcomes for which data were sought. Specify whether all results that were compatible with each outcome domain in each study were sought (e.g. for all measures, time points, analyses), and if not, the methods used to decide which results to collect. | Page 3 |
|  | 10b | List and define all other variables for which data were sought (e.g. participant and intervention characteristics, funding sources). Describe any assumptions made about any missing or unclear information. | Page 3 |
| Study risk of bias assessment | 11 | Specify the methods used to assess risk of bias in the included studies, including details of the tool(s) used, how many reviewers assessed each study and whether they worked independently, and if applicable, details of automation tools used in the process. | Page 4 |
| Effect measures | 12 | Specify for each outcome the effect measure(s) (e.g. risk ratio, mean difference) used in the synthesis or presentation of results. | Page 4 |
| Synthesis methods | 13a | Describe the processes used to decide which studies were eligible for each synthesis (e.g. tabulating the study intervention characteristics and comparing against the planned groups for each synthesis (item #5)). | Page 4 |
|  | 13b | Describe any methods required to prepare the data for presentation or synthesis, such as handling of missing summary statistics, or data conversions. | Page 3 |
|  | 13c | Describe any methods used to tabulate or visually display results of individual studies and syntheses. | Page 3, 4 |
|  | 13d | Describe any methods used to synthesize results and provide a rationale for the choice(s). If meta-analysis was performed, describe the model(s), method(s) to identify the presence and extent of statistical heterogeneity, and software package(s) used. | Page 4 |
|  | 13e | Describe any methods used to explore possible causes of heterogeneity among study results (e.g. subgroup analysis, meta-regression). | Page 4 |
|  | 13f | Describe any sensitivity analyses conducted to assess robustness of the synthesized results. | Page 4 |
| Reporting bias assessment | 14 | Describe any methods used to assess risk of bias due to missing results in a synthesis (arising from reporting biases). | Page 4 |
| Certainty assessment | 15 | Describe any methods used to assess certainty (or confidence) in the body of evidence for an outcome. | Page 4 |
| **RESULTS** | | |  |
| Study selection | 16a | Describe the results of the search and selection process, from the number of records identified in the search to the number of studies included in the review, ideally using a flow diagram. | Page 4, 5 |
|  | 16b | Cite studies that might appear to meet the inclusion criteria, but which were excluded, and explain why they were excluded. | Page 4 |
| Study characteristics | 17 | Cite each included study and present its characteristics. | Page 4, 5 Table 1 |
| Risk of bias in studies | 18 | Present assessments of risk of bias for each included study. | Page 13 |
| Results of individual studies | 19 | For all outcomes, present, for each study: (a) summary statistics for each group (where appropriate) and (b) an effect estimate and its precision (e.g. confidence/credible interval), ideally using structured tables or plots. | Page 13-16  Tables 1-3  Figure 2 |
| Results of syntheses | 20a | For each synthesis, briefly summarise the characteristics and risk of bias among contributing studies. | Page 13-16 |
|  | 20b | Present results of all statistical syntheses conducted. If meta-analysis was done, present for each the summary estimate and its precision (e.g. confidence/credible interval) and measures of statistical heterogeneity. If comparing groups, describe the direction of the effect. | Page 13-16 |
|  | 20c | Present results of all investigations of possible causes of heterogeneity among study results. | Page 13 |
|  | 20d | Present results of all sensitivity analyses conducted to assess the robustness of the synthesized results. | Page 13 |
| Reporting biases | 21 | Present assessments of risk of bias due to missing results (arising from reporting biases) for each synthesis assessed. | Page 13 |
| Certainty of evidence | 22 | Present assessments of certainty (or confidence) in the body of evidence for each outcome assessed. | Page 13-16 |
| **DISCUSSION** | | |  |
| Discussion | 23a | Provide a general interpretation of the results in the context of other evidence. | Page 16 |
|  | 23b | Discuss any limitations of the evidence included in the review. | Page 17, 18 |
|  | 23c | Discuss any limitations of the review processes used. | Page 17, 18 |
|  | 23d | Discuss implications of the results for practice, policy, and future research. | Page 17 |
| **OTHER INFORMATION** | | |  |
| Registration and protocol | 24a | Provide registration information for the review, including register name and registration number, or state that the review was not registered. | Page 2 |
|  | 24b | Indicate where the review protocol can be accessed, or state that a protocol was not prepared. | Page 2 |
|  | 24c | Describe and explain any amendments to information provided at registration or in the protocol. | NA |
| Support | 25 | Describe sources of financial or non-financial support for the review, and the role of the funders or sponsors in the review. | Page 18 |
| Competing interests | 26 | Declare any competing interests of review authors. | Page 18 |
| Availability of data, code and other materials | 27 | Report which of the following are publicly available and where they can be found: template data collection forms; data extracted from included studies; data used for all analyses; analytic code; any other materials used in the review. | Page 18 |

*From:*  Page MJ, McKenzie JE, Bossuyt PM, Boutron I, Hoffmann TC, Mulrow CD, et al. The PRISMA 2020 statement: an updated guideline for reporting systematic reviews. BMJ 2021;372:n71. doi: 10.1136/bmj.n71. This work is licensed under CC BY 4.0. To view a copy of this license, visit <https://creativecommons.org/licenses/by/4.0/>

# Appendix K: PRISMA for abstract checklist

| **Section and Topic** | **Item #** | **Checklist item** | **Reported (Yes/No)** |
| --- | --- | --- | --- |
| **TITLE** | | |  |
| Title | 1 | Identify the report as a systematic review. | Yes |
| **BACKGROUND** | | |  |
| Objectives | 2 | Provide an explicit statement of the main objective(s) or question(s) the review addresses. | Yes |
| **METHODS** | | |  |
| Eligibility criteria | 3 | Specify the inclusion and exclusion criteria for the review. | Yes |
| Information sources | 4 | Specify the information sources (e.g. databases, registers) used to identify studies and the date when each was last searched. | Yes |
| Risk of bias | 5 | Specify the methods used to assess risk of bias in the included studies. | Yes |
| Synthesis of results | 6 | Specify the methods used to present and synthesise results. | Yes |
| **RESULTS** | | |  |
| Included studies | 7 | Give the total number of included studies and participants and summarise relevant characteristics of studies. | Yes |
| Synthesis of results | 8 | Present results for main outcomes, preferably indicating the number of included studies and participants for each. If meta-analysis was done, report the summary estimate and confidence/credible interval. If comparing groups, indicate the direction of the effect (i.e. which group is favoured). | Yes |
| **DISCUSSION** | | |  |
| Limitations of evidence | 9 | Provide a brief summary of the limitations of the evidence included in the review (e.g. study risk of bias, inconsistency and imprecision). |  |
| Interpretation | 10 | Provide a general interpretation of the results and important implications. |  |
| **OTHER** | | |  |
| Funding | 11 | Specify the primary source of funding for the review. |  |
| Registration | 12 | Provide the register name and registration number. |  |

*From:*  Page MJ, McKenzie JE, Bossuyt PM, Boutron I, Hoffmann TC, Mulrow CD, et al. The PRISMA 2020 statement: an updated guideline for reporting systematic reviews. BMJ 2021;372:n71. doi: 10.1136/bmj.n71. This work is licensed under CC BY 4.0. To view a copy of this license, visit <https://creativecommons.org/licenses/by/4.0/>
